# How Swimming Modulates Inflammatory Pathways in Pain, Neurodegenerative, and Metabolic Disorders

**DOI:** 10.3390/brainsci15101121

**Published:** 2025-10-18

**Authors:** Mahdiyeh Kooshki, Rozhin Rezeai-Farimani, Amirmohammad Moradpour, Vafa Baradaran Rahimi, Vahid Reza Askari

**Affiliations:** 1 Patient Safety Research Center, Clinical Research Institute, Mashhad University of Medical Sciences, Mashhad 9177948564, Iran; mkooshki77@yahoo.com (M.K.); am.moradpour@yahoo.com (A.M.); vafa_br@yahoo.com (V.B.R.); 2 Transplant Research Center, Clinical Research Institute, Mashhad University of Medical Sciences, Mashhad 9177948564, Iran; rozhiiinrezaei83@gmail.com; 3 Student Research Committee, Mashhad University of Medical Sciences, Mashhad 9177948564, Iran; 4 Clinical Research Development Unit, Imam Reza Hospital, Faculty of Medicine, Mashhad University of Medical Sciences, Azadi Sq, Vakil Abad Highway, Mashhad 9177948564, Iran; 5 Department of Cardiovascular Diseases, Faculty of Medicine, Mashhad University of Medical Sciences, Mashhad 9177948564, Iran

**Keywords:** inflammation, Alzheimer’s disease, osteoarthritis, rheumatoid arthritis, cardiovascular disorders

## Abstract

**Background:** As a non-weight-bearing full-body exercise, swimming may reduce inflammation and boost anti-inflammatory agents to decrease the risk of cardiovascular, neurological, and rheumatological disorders. This systematic review examines the current evidence on the role of swimming exercise in modulating immune responses through inflammatory pathways. **Methods:** First, the PubMed and Scopus databases were searched through December 2024 for studies on swimming and inflammation. The initial search using keywords yielded 509 articles; 102 met the inclusion criteria after screening for relevance, language, and full-text availability. **Results:** This study suggests that regular swimming reduces neuroinflammation by enhancing BDNF, CREB, and PI3K/Akt signaling while suppressing both mRNA and protein levels of NF-κB, TNF-α, and IL-6 in the brain. In metabolic tissues, it activates SIRT1 and PGC-1α, improving mitochondrial biogenesis and antioxidant defense. Swimming also upregulates PPAR-α and eNOS while downregulating iNOS, leading to reduced vascular inflammation, oxidative stress, and fibrosis in renal and cardiac tissues. Moreover, the enhanced production of IL-10 and the decreasing levels of IL-1β and CRP contribute to systemic anti-inflammatory effects. **Conclusions:** Consequently, the available evidence suggests that swimming can be a low-impact, full-body exercise with potential therapeutic options in managing inflammation-related conditions such as cardiovascular disease, diabetes, and obesity. Future studies should focus on human clinical trials, investigate mechanisms, and assess longer time frames.

## 1. Introduction

### 1.1. Swimming

Physical activity, particularly swimming, has a significant influence on the overall health of humans, as well as their physical and cellular growth, and positively impacts cognitive function regulation [1]. Meta-analytic evidence demonstrates that swimming uniquely improves lipid profiles—reducing total cholesterol by 0.31 mmol/L (*p* = 0.004) and increasing HDL cholesterol by 0.15 mmol/L (*p* < 0.001)—alongside reducing body fat percentage by 2.98% (*p* < 0.001) and all-cause mortality by 24% (HR = 0.76; 95% CI: 0.63, 0.92), whereas running shows no significant lipid improvements despite superior cardiorespiratory gains (VO_2max_ + 5.75 mL/kg/min, *p* < 0.001) [2]. Swimming has emerged as a widely practiced sport globally due to its aquatic environment and numerous health benefits. A notable advantage of swimming is its low impact on bones and joints due to the buoyancy of water. However, high-intensity training regimens may result in joint and muscular overload, thereby elevating the risk of injury. The most frequently observed swimming injuries primarily affect the athlete’s knee, lumbar/hip region, and shoulder [3]. However, possibly due to the difficulty of this sport, it has gained less attention compared to running or cycling in the scientific literature. Swimming exercise has been observed to modulate immune and endocrine function, potentially through the reduction of inflammatory cytokines and corticosterone levels, as well as the elevation of testosterone concentrations [4].

### 1.2. Swimming in Medical Sciences

In order to assess the impact of swimming on inflammation, studies mainly rely on small rodent animals, such as rats and mice [5]. Both clinical and animal studies support the connection between excessive inflammation and depressive disorder [4]. Empirical evidence indicates that consistent engagement in physical exercise may attenuate oxidative stress (OS) and facilitate the upregulation of anti-inflammatory agents and a host of enzymes that mitigate inflammation [6]. Evidence from multiple studies shows that physical activity lowers inflammation and glutamate-induced neurotoxicity, as well as the development of the neurotrophic system. Additionally, some experimental records indicate that strength training may have a promising impact on memory function [5]. Regular physical exercise, even when initiated in later life, may potentially delay the onset of diseases and functional decline associated with the aging process. This effect is hypothesized to occur by attenuating potentially detrimental oxidative damage and suppressing inflammatory processes [7].

### 1.3. Inflammation and Inflammatory Diseases

Dysregulated oxidant production leads to oxidative stress (OS), resulting in the excessive generation of reactive oxygen species (ROS) [8]. This process impairs cellular function, promotes cancerous diseases, and leads to toxic levels of harmful compounds [8,9]. Research indicates a correlation between elevated ROS levels and various neurological disorders, particularly those that degenerate neurons, such as Parkinson’s disease (PD) [10] and Alzheimer’s disease (AD), as well as seizures [11]. The hippocampus is mainly sensitive to the detrimental effects of oxidative damage. Evidence suggests that excessive ROS generation promotes the synthesis of pro-inflammatory cytokines [12,13,14]. Inflammation is a common complication in various conditions, including painful diseases such as gout, pseudogout, and osteoarthritis [15,16,17]. Muscular pain is a ubiquitous phenomenon associated with diverse pathological conditions, including cervical and scapular discomfort, non-specific lumbar pain, and myofascial pain syndrome.

Notwithstanding the advent of diverse therapeutic modalities—ranging from pharmacological interventions utilizing non-steroidal anti-inflammatory agents to physical therapy and dry needling—a universally efficacious strategy for pain management has yet to be established [15]. Muscle fiber degradation resulting from overuse and chronic inadequate blood perfusion leads to a localized reduction in pH. The inflammation caused by these processes contributes to the manifestation of muscle pain [15]. Unfortunately, effective therapeutic strategies are currently limited [18], and medications that are currently available on the market have adverse effects [19].

Exercise may lead to a reduction in symptoms of depression through mitigating inflammation [20]. The existing literature suggests that aerobic activities may enhance the expression of synaptic plasticity-associated proteins by reducing the release of nuclear factor kappa light chain enhancer of activated B cells (NF-κB) and interleukin-1 beta (IL-1β) in hippocampal regions. This evidence implies that aerobic exercise can potentially ameliorate neuroinflammation, consequently improving cognition [1]. Sirtuin 1 (SIRT1), a nicotinamide adenine dinucleotide (NAD^+^)-dependent class III histone deacetylase enzyme, plays a role in cellular aging, metabolic homeostasis, inflammation of neural cells, as well as aging processes through the activation of proliferator-activated receptor gamma coactivator-1 alpha (PGC-1α)/brain-derived neurotrophic factor (BDNF) and disruption of the NF-κB pathway. This enzymatic entity catalyzes the deacetylation of histones in the promoter region of NF-κB-p65, thereby attenuating the secretion of pro-inflammatory cytokines such as tumor necrosis factor-alpha (TNF-α) and IL-1β, and mitigating the inflammatory cascades. An expanding body of evidence suggests that being physically active can ameliorate cognitive deficits via SIRT1-mediated signaling pathways [1].

Given the limited research on swimming interventions, this systematic review aims to examine their effects on inflammatory and anti-inflammatory factors.

## 2. Methods

This review follows the Preferred Reporting Items for Systematic Reviews and Meta-Analyses (PRISMA). The methodology section delineates the search methods for study identification and the criteria for study inclusion. Electronic databases, specifically PubMed and Scopus, were systematically searched from the inception to the end of 2024. Eligibility was limited to English-language articles from which relevant data were subsequently extracted.

The initial literature search, utilizing the keyword “swimming” concerning inflammatory diseases, yielded 509 articles (detailed in Table 1). After duplicates were eliminated and the titles, abstracts, and whole manuscripts were checked, 102 articles met the eligibility criteria. A total of 407 studies were excluded for various reasons, including duplication, irrelevance, and inaccessibility of full-text articles. The number of articles obtained by searching for different words, along with the words swimming and inflammation in various databases, is presented in Table 1. 

## 3. Results and Discussion

### 3.1. Neurological Disorders

Dysregulation of the hippocampus and prefrontal cortex can cause significant changes in working, spatial, and recognition memory. Cognitive deterioration is correlated with multiple neurobiological alterations, encompassing an upregulation of pro-inflammatory cytokines, notably TNF-α, IL-1β, and IL-6; a concomitant downregulation of BDNF and cytokine IL-10; and neurotoxicity caused by excessive glutamate release under inflammatory conditions [9,10,11,13,17,21]. Research suggests that elevated levels of inflammatory cytokines in the prefrontal cortex and the hippocampus may trigger the characteristic symptoms of depression [4]. Stress has been observed to activate microglial cells, leading to the subsequent release of pro-inflammatory factors. This inflammatory response is associated with the manifestation of depressive and anxiety-like symptoms [12,22]. Nuclear factor erythroid 2-related factor 2 (Nrf2) has been shown to lower inflammation and has a major role in mitigating the damage caused by OS. It contributes to suppressing inflammation by dampening the production of pro-inflammatory cytokines and NF-κB cascades. Furthermore, activation of this factor has been observed to enhance the levels of BDNF by downregulating its transcriptional repressors [12].

#### 3.1.1. Alzheimer’s Disease (AD)

In a study, Liu et al. (2024) [23] investigated D-galactose model mice subjected to an eight-week exercise regimen (Table 2). Their findings elucidated a diminution in inflammation associated with astrocytic secretions, specifically C-X-C motif chemokine ligand 2, cyclooxygenase-2, and TNF-α. Moreover, they reported decreased expression of microRNA-34a (miR-34a), accompanied by activation of the cyclic adenosine monophosphate (cAMP) response element-binding protein (CREB)/phosphoinositide 3-kinase (PI3K)/TATA-box binding protein-associated factor 1 signaling cascade. The investigators suggested that treatment with physical activity lowers miR-34a and potentially protects neurons against damage by mitigating the loss of myelin and the perfusion of pro-inflammatory agents into the central nervous system (CNS) [23].

Farsani et al. (2024) [21] assessed the impact of strength training on profilin-1 (PFN1), semaphorin 3A (SEMA3A), and neural cell adhesion molecule (NCAM) in the gastrocnemius muscle, showing AD-like symptoms. The study utilized 32 male Wistar rats (6 weeks old), inducing AD-like phenotypes through the injection of beta-amyloid into the hippocampus (Table 2). Following 20 swimming sessions, the researchers observed:

- There was an increase in SEMA3A and a decrease in PFN1 and NCAM in the AD-like control group. The opposite trend was observed in the healthy controls.
- There was a reduction in SEMA3A and an increase in NCAM and PFN1 in the healthy trained (HT) group compared to the healthy controls.

It was concluded that the regeneration of axons and, therefore, neuronal formation in motor neurons can be effectively altered as a result of swimming [21].

Additionally, in 2023, Karaji et al. [24] investigated the effects of swimming exercise and clove supplementation on memory function, dark cell populations, and the expression of α7nicotinic acetylcholine receptor (α7nAChR) and NLR family pyrin domain containing 1 (NLRP1) in the hippocampus of a rat model of AD. The study involved 48 rodents, with AD induced via amyloid β injection. The experimental protocol included a three-week swimming regimen and daily clove supplementation. The results showed that both interventions attenuated AD-induced changes in α7nAChR expression, NLRP1 expression, memory function, and dark cell population, suggesting potential therapeutic benefits in mitigating hippocampal changes associated with AD in this model.

Liu et al. (2022) [25] investigated how a 4-week aerobic interval exercise regimen, alternating between running and swimming, affects cognition in an adult neurogenesis mouse model of AD. The study used Morris’s water maze assessments to demonstrate that AD mice in the treatment group exhibited enhanced cognitive performance relative to sedentary controls. Biochemical analyses revealed that the treatment group had elevated concentrations of B-cell lymphoma-2 (Bcl-2), postsynaptic density 95 (PSD95), synaptophysin, and neuronal nuclei (NeuN), as well as reduced expression of glial fibrillary acidic protein (GFAP). The investigators suggested that the exercise-mediated alterations in proteins and metabolism contributed to the augmented growth of neurons and improved outcomes in the context of AD pathology, suggesting the potential therapeutic efficacy of aerobic interval exercise in attenuating AD-associated cognitive decline [25].

Hegazy et al. (2022) [26] compared the effects of swimming and L-carnosine supplementation on serum, hippocampal, and cerebrospinal fluid (CSF) levels of irisin following treatment with streptozotocin (STZ). The study found reduced hippocampal irisin concentrations after intracerebroventricular (ICV) STZ administration, but not in CSF or serum. Following a 5-week swimming regimen and daily oral carnosine (100 mg/kg/day), both interventions normalized hippocampal fibronectin type III domain-containing protein 5 (FNDC5)/irisin expression. This normalization was linked to reduced soluble β-amyloid and phosphorylated tau protein levels, enhanced BDNF and insulin signaling proteins, and improved cognitive function. The study concluded that carnosine supplementation was as effective as exercise in reversing cognitive decline and AD-related biomarkers, potentially through enhancing hippocampal FNDC5/irisin and insulin signaling [26].

Bashiri et al. (2020) [27] studied how swimming affects cognitive function, behaviors associated with anxiety, and symptoms of depression in rat models of AD. The researchers induced an AD model in mice using STZ injections, followed by a four-week swimming regimen initiated eight days post-injection. Results indicated that swimming exercise significantly mitigated STZ-induced neurobehavioral disorders, as reflected by higher BDNF levels and reduced concentrations of both glutamate and TNF-α in the hippocampus of mice treated with STZ. Based on these results, the authors stated that swimming correlates with a reduced incidence of neurobehavioral diseases in individuals with AD. However, more studies are necessary to confirm these results and explore potential clinical applications [27].

**Figure 1:**
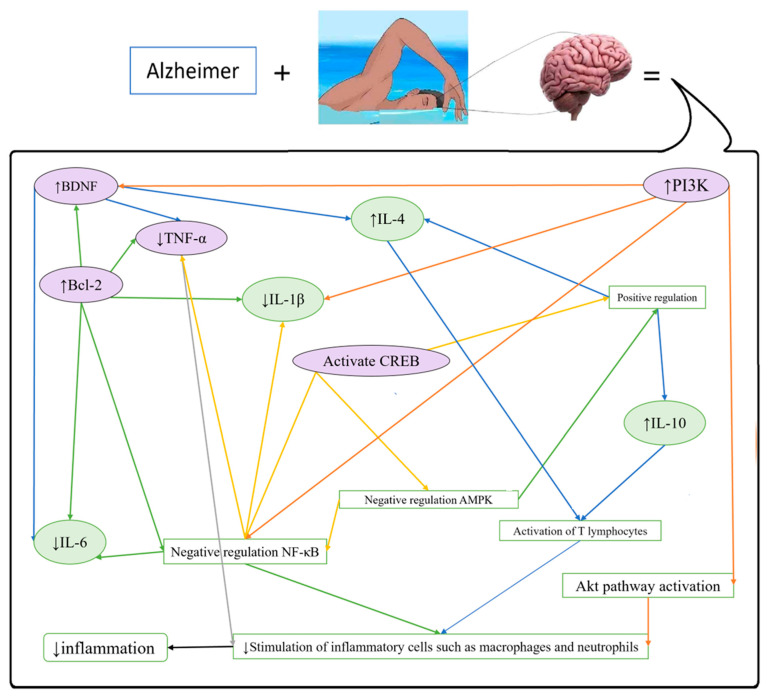
The effect of regular swimming practice on inflammation in Alzheimer’s patients. Regular swimming exercises in Alzheimer’s patients activate the CREB pathway, reduce TNF-α levels, and increase the expression of PI3K, Bcl-2, and BDNF. These changes activate the Akt pathway, increase IL-4 and IL-10, and negatively regulate NF-κB and AMPK, thereby reducing the stimulation of macrophages and neutrophils and ultimately decreasing inflammation. Abbreviations: AMP-activated protein kinase (AMPK); B-cell lymphoma-2 (Bcl-2); brain-derived neurotrophic factor (BDNF); cAMP response element-binding protein (CREB); interleukin-4 (IL-4); interleukin-10 (IL-10); nuclear factor kappa light-chain-enhancer of activated B lymphocytes (NF-κB); phosphoinositide 3-kinase (PI3K); protein kinase B (Akt); tumor necrosis factor-alpha (TNF-α); ↑: increase; ↓: decrease.

Wu et al. (2018) [28] examined the potential impact of exercise as a pretreatment in a sporadic AD rat model. The study utilized 2.5-month-old male Sprague Dawley rats. STZ injection was found to increase several markers in the hippocampus, especially the CA1 region, including reactive gliosis, proinflammatory cytokine release, oxidative damage, amyloid beta levels, tau hyperphosphorylation, Nrf2 protein expression, DNA binding activity, and downstream antioxidant gene expression. Following exercise intervention, these effects were significantly attenuated. Histological analysis revealed that exercise-induced substantial neuroprotection and suppressed neuronal apoptotic-like cell death in the hippocampal CA1 region, in contrast to the STZ control group. These findings suggest a potential protective effect of exercise in this AD model, although further research would be necessary to confirm and extend these results (Figure 1) [28].

#### 3.1.2. Parkinson’s Disease (PD)

Gergin et al. (2023) [29] investigated combined treatment with melatonin and frequent swimming exercise in a 6-hydroxydopamine (6-OHDA)-induced rat model of PD, focusing on the shape of the dendritic spine in striatal neurons (Table 2). The study utilized 24 male Wistar albino rats. Following PD model induction, the subjects (*Rattus norvegicus*) were subjected to a six-week moderate-intensity swimming protocol. This regimen consisted of 30 min sessions conducted five times per week, preceded by a two-week acclimatization period. Subsequent analysis revealed a statistically significant increase in calretinin-positive interneurons within the striatum of the PD model rats that underwent the swimming protocol. Both the swimming intervention and melatonin administration were observed to attenuate the reduction in overall dendritic spine density, with particular preservation of mushroom-type spines [29].

Che Ramli et al. (2022) [19] evaluated the impact of *Bacopa monnieri* extract on rotenone-induced PD in 50 adult zebrafish. The researchers observed increased swimming activity in treated fish, which they attributed to the potential antioxidant and therapeutic properties of *Bacopa monnieri*. Based on these findings, the authors suggest that *Bacopa monnieri* extract could potentially be considered for treating PD in this zebrafish model [19].

Skripkina et al. (2021) [30] examined the deterioration of swimming abilities in individuals with Parkinson’s disorder. The investigation identified three primary factors contributing to impaired swimming performance in this population: motor difficulties affecting limb movement, deficits in coordination, and the inability to maintain proper horizontal positioning while swimming [30]. Another study, conducted in 2021, aimed to evaluate the swimming ability of patients with PD, reporting that swimming ability is impaired in a subset of individuals with PD. This finding suggests variability in swimming performance among PD patients, with some experiencing compromised aquatic motor skills [31].

Notably, Boracı and colleagues (2020) [32] evaluated the potential neuroprotective impact of frequent physical activity on striatal calretinin-positive interneurons in a rat model of PD. The investigation employed Wistar rats with PD-like symptoms induced by 6-OHDA injections. Subjects in the exercise cohort participated in daily swimming sessions lasting 30 min over a 6-week period. Apomorphine-induced rotation assessments were performed at weeks 3 and 6. Upon completion of the intervention, brain tissue was harvested following transcardiac perfusion. The findings demonstrated a statistically significant elevation in calretinin-positive interneurons within the striatum of subjects in the treatment group, accompanied by a considerable decrease in rotational behavior. Based on these observations, the investigators proposed that striatal calretinin-positive interneurons may contribute substantially to the neuroprotective mechanisms associated with exercise in the context of PD [32].

**Table 2:** Summary of results from In Vivo animal studies.

Row	Type of Disease	Inducing Agent	Intervention	Results	Reference
1	Alzheimer’s Disease (AD)	D-galactose intraperitoneally (i.p.)	Exercise (EXE) for 8 weeks	TATA-box binding protein-associated factor 1/phosphoinositide 3-kinase/cyclic adenosine monophosphate response element-binding protein signaling pathway activation ↑ Number of neuronal nuclei (NeuN^+^) neurons by ↓ excessive demyelination ↑ Expression of myelin basic protein ↑ Expression of oligodendrocyte markers, oligodendrocyte transcription factor 2, and platelet-derived growth factor receptor alpha related to brain proliferation ↑ Spatial cognitive performance ↓ Inflammatory responses resulted from astrocyte secretions (tumor necrosis factor alpha [TNF-α], cyclooxygenase-2, C-X-C motif chemokine ligand 2) ↓ Levels of miR-34a	[23]
2	AD	Hippocampus injection of β-amyloid peptide (Aβ)	Swimming (20 sessions)	↑ Neural cell adhesion molecule and profilin-1 ↓ Semaphorin 3A ↑ Axon reformation and neuronal generation in motor neurons	[21]
3	AD	Aβ (stereotaxic intracerebral injection)	Swimming training (ST) (30 min a day, 3 weeks) daily clove supplement (0.1 mg/kg) gavage	↑ α7nicotinic acetylcholine receptor ↓ NLR family pyrin domain-containing 1 and dark cells	[24]
4	AD	Aβ oligomer	EXE (4 weeks)	↓ Anxiety and depression-like behaviors (ADB) ↑ Newborn cells (mostly differentiated into neurons, bromodeoxyuridine [BrdU^+^]DCX^+^ cells or BrdU^+^NeuN^+^ cells, and a few astrocytes, BrdU^+^ glial fibrillary acidic protein (GFAP^+^) cells) ↑ NeuN, postsynaptic density 95, synaptophysin, B-cell lymphoma-2 (Bcl-2) ↓ GFAP protein	[25]
5	AD	Intracerebroventricular (ICV) injection of streptozotocin (STZ)	ST (5 weeks) Oral carnosine (100 mg/kg/day)	↑ Hippocampal fibronectin type III domain-containing protein 5/irisin expression by ↓ Soluble β-amyloid peptide ↓ Phosphorylated tau protein, ↑ Brain-derived neurotrophic factor and insulin signaling proteins, ↓ Cognitive impairments	[26]
6	AD	STZ (i.p.)	ST (4 weeks)	↓ Cognitive functions impairing ↓ ADB, ↓ Aβ-42, glutamate, malondialdehyde (MDA), TNF-α, and interleukin-6 (IL-6) levels in the hippocampus ↑ BDNF	[27]
7	AD	Aluminium chloride (70 mg/kg, i.p.)	Vinpocetine (20 mg/kg, p.o.) Coenzyme Q10 (200 mg/kg, orally) Swimming and Y-maze tests once per week for four weeks	↓ MDA ↑ Superoxide dismutase (SOD) and total antioxidant capacity (TOC) levels ↓ Lipid peroxidation Improved the antioxidant status	[33]
8	Neuropathies	Brachial plexus avulsion (BPA) surgery is performed on the unilateral brachial plexus.	After BPA surgery Cold-water swimming or sham training for 5 min twice a day for a period of two weeks	↑ Grip force induced ↓ Threshold of paw withdrawal responses ↓ Expression of substance P and ionized calcium-binding adaptor molecule 1 in dorsal horns, especially in Lamina I and II	[34]
9	Neuropathies	Nerve compression for 30 s using hemostatic forceps	Sericin silk protein (hydrolyzed sericin is applied directly to the injury in the injury-sericin and injury-sericin-swimming groups). Swimming 5 days per week for 3 weeks, with a 10% overload of the body weight (weekly progressive elevation, lasting 15, 20, and 25 min/day).	Enhancement in injury-swimming performance relative to other groups ↑ Fibers of smaller diameter in the injury-sericin-swimming ↑ Improvement of the nociceptive threshold and allodynia by swimming Exacerbated pro-inflammatory responses by Sericin treatment	[35]
10	Parkinson	6-Hydroxydopamine (6-OHDA) was injected unilaterally	Melatonin ST at moderate intensity (30 min, 5 times a week, 6 weeks)	Swimming EXE: striking elevation of calretinin-positive interneurons in the striatum Swimming EXE + melatonin: preserved the loss of spine density and the loss of spine density of mushroom-type spines	[29]
11	Parkinson	6-OHDA (injected unilaterally into the medial forebrain bundle)	Daily ST (30 min, 6 weeks)	↑ Calretinin-positive interneurons ↓ Number of rotations	[32]
12	Parkinson	6-OHDA (stereotaxic intracerebral injection)	ST (4 weeks)	It attenuates the following impairments as a consequence of 6-OHDA exposure: (i) Depressive-like behavior in the tail suspension test (ii) ↑ The number of falls in the rotarod test; (iii) Impairment of chronic memory in the object recognition test (iv) ↑ Reactive species and interleukin-1 beta (IL-1β) levels (v) Inhibition of the Glutathione peroxidase activity (vi) Elevation of the glutathione ↓ Glutathione reductase (GR) and glutathione S-transferase activities (vii) ↓ Dopamine (DA), homo vanillic acid, and 3,4- 3,4-dihydroxyphenylacetic acid levels. ↓ Cognitive and motor declines, depression, oxidative stress (OS), and neuroinflammation induced by 6-OHDA	[36]
13	Osteoarthritis	Mouse model of aged knee osteoarthritis (OA) through natural aging of mice	Adaptive swimming for 1 week and formal swimming for 8 weeks (15 min, once a day, 3 days a week, for a total period of 8 weeks)	The mice in the aged group, compared with the young group, showed ↓ Stride length ↑ Number of peripheral leukocytes and lymphocytes ↓ Count of chondrocytes ↑ Modified Mankin’s score ↓ Protein and mRNA expression of type II collagen and aggrecan ↑ Matrix metalloproteinase-13 (MMP-13) expression ↓ Proteoglycan In comparison to the aged group, ST remarkably enhanced the stride length of mice ↓ Count of peripheral blood lymphocytes ↑ Count of chondrocytes ↓ Modified Mankin’s score ↑ Protein and mRNA expression of type II collagen and aggrecan ↓ Expression of matrix metalloproteinase The articular surface is smooth, chondrocytes are normal, and proteoglycan loss is less. ↓ Number of inflammatory cells in the blood Enhanced articular chondrocytes, matrix composition, and cartilage tissue morphology.	[37]
14	Osteoarthritis	100 μL monosodium iodoacetate was injected intra-articularly into the right knee	Oral saline (4 weeks) ST (20 min) Oral curcumin 200 mg/kg till the end of the experimental period (35 days).	Swimming caused: ↓ Pain and joint stiffness Development of histological and radiological osteoarthritis occurrence in the knee joints ↓ Serum C-reactive protein (CRP) and tissue cartilage oligomeric matrix protein levels Restoring the miR-130a and histone deacetylase 3 The dual therapy caused: Increased peroxisome proliferator-activated receptor gamma (PPAR-γ) alongside ↓ nuclear factor kappa light-chain-enhancer of activated B lymphocytes (NF-κB) and its inflammatory cytokine targets TNF-α and IL-1β. ↓ Matrix metalloproteinase-1 and MMP-13	[38]
15	Osteoarthritis	100 mL of complete Freund’s adjuvant (CFA), inactivated *M. butyricum* (1 mg/mL), or 100 mL of saline (control group), was injected into the intra-articular space of the left ankle.	The stimulation of electroacupuncture (EA) was utilized at acupoints ST36 and GB30 on the left ankle (5 times a week, lasting for 30 min) The protocol consists of 10 min of swimming three times a week. The EA + swimming group received different EA sessions, three times a week swimming, and twice a week EA sessions.	EA group: Decreased the nociceptive scores ↑Latency time in thermal cold and heat hyperalgesia. ↓ *N*-acetyl-β-D-glucosaminidase. Swimming group: ↓ Edema Did not elevate the inflammatory infiltrates or congestion EA + swimming: ↓ Neutrophils, monocytes, myeloperoxidase, and glutamate levels in the cerebrospinal fluid	[39]
16	Osteoarthritis	Median meniscectomy	Treadmill protocol: In weeks 1 and 2, the running time was 30 min with a 13 m/min speed, without incline. The execution time in week 3 was 30 min, and in weeks 4, 5, and 6 was 50 min. In weeks 3, 4, 5, and 6, the speed was 16 m/min. Training intensities and volumes were light to moderate (50% and 60% of maximal oxygen uptake [VO_2max_]). Swimming protocol: The adaptation period was 20 min per day for 1 week. A swimming program, consisting of 6 weeks and 20 min of daily sessions, has been conducted in alternating sessions on weekdays.	The treadmill was more effective ○ ↓ Pro-inflammatory cytokines (Interferon gamma, TNF-α, IL-1β, and IL-6) ○ ↑ Anti-inflammatories (interleukin-4 [IL-4], interleukin-10 [IL-10], and transforming growth factor beta [TGF-β]) ○ Sustaining a more controlled oxi-reductive environment within the joint, ○ Provided a more satisfactory morphological result in terms of the number of chondrocytes in the histological evaluation.	[40]
17	Osteoarthritis	Injection of papain in the knees: a total of 0.2 mL of 4% papain solution, 0.1 mL of 0.03 M cysteine (activator) was injected intra-articularly with a microsyringe into the right knee of the animal	All treatments were initiated 21 days after the final papain injection and were administered once daily, three times a week (every other day), for eight consecutive weeks, totaling 24 treatment sessions. Sodium diclofenac gel (10 mg∕knee applied) Swimming EXE (2 weeks adapt + 6 weeks training) PBMT: using a laser diode with a wavelength of 830 nm (infrared) continuously, spot area of 0.028 cm^2^, power of 100 mW, power density of 35.71 W∕cm^2^, energy density of 214.2 J∕cm^2^, energy of 6 J per point, 60 s per point, and 1 point on the OA joint.	Photobiomodulation therapy (PBMT) and nonsteroidal anti-inflammatory drugs reduced the total amount of cells in the inflammatory infiltrate, and PBMT was the most effective for reducing the activity of myeloperoxidase PBMT was the most impactful therapy in stopping disease development and enhancing inflammatory conditions observed in OA	[41]
18	Rheumatoid Arthritis	Age (young: 4 weeks; middle-aged: 14 months; rats)	ST for 9 weeks	Improved cognitive functions in both groups with a parallel ○ ↓ Protein carbonyls of the brain. ○ ↑ Proteasome activity by the EXE in skeletal muscle. ○ ↑ In the amount of NF-κB, the amount of inhibitor of kappa B in the cytoplasm was higher in the middle-aged animals. Eight weeks of regular exercise are able to reverse the decrease in the activity of GR in the liver. Increase the serum level of glucocorticoids in the exercised old animals.	[7]
19	Rheumatoid Arthritis	Bovine type II collagen (1 mg/mL) was injected into the right hind paw of each animal	ST (6 weeks, 5 days/week, 60 min/day)	↑ lymphocyte proliferation and macrophage H_2_O_2_ production Prevent the activation of immune cells induced by collagen-induced arthritis ↑ Plasma levels of corticosterone, progesterone, and interleukin-2 (IL-2).	[42]
20	Gout	Oxonic acid (1 mL/kg) and hypoxanthine (administered via diet)	Swimming in cold water at 10–12 °C, 10 min every day, for 12 days	↑ The uric acid concentration in the joint cavity ↑ Uric acid in hyperuricemia + swimming in cold water Ankle injuries, such as hemangiectasis, congestion, and infiltration of inflammatory cells in the synovium and the soft tissue around, in the hyperuricemia + swimming in cold water group	[43]
21	Renal Disorders	Age (21 months old)	12 weeks of moderate swimming exercise in aged rats (21 months old)	↓ Plasma levels of creatinine and blood urea nitrogen ↓ Renal injuries (fibrosis, OS, triglyceride levels, and the mRNA expression of actin alpha 2, Fn, collagen type I alpha 1, type IV collagen alpha chain, and TGF-β1. ↓ OS (↓ MDA level and ↑ manganese superoxide dismutase activity in kidneys). Inhibited NF-κB activities ↓ Renal expression of pro-inflammatory cytokines, including monocyte chemoattractant protein-1, IL-1β, and IL-6. Activates peroxisome proliferator-activated receptor alpha (PPAR-α)	[44]
22	Renal Disorders	High-sodium diet (Addition of NaCl up to 2% *w*/*w* yielding a 0.90% *w*/*w* Na + chow)	4-day adaptation period (15 min swimming on the first day and 15 min elevation in swimming time each day until reaching 60 min on the fourth day). From the second week on, training was done in the morning period, from 09:00 to 11:00 h, 5 days/week for 2 h straight and free of loading for 9 weeks	↓ Blood pressure (BP) ↓ Cerebrospinal fluid (CSF) [Na+] and renal fibrosis	[45]
23	Renal Disorders	Doxorubicin injection (i.p.)	ST Garlic extract	Swimming alone or supplemented with garlic extract ↓ MDA value Improved the activities of antioxidant enzymes ↓ Blood alanine aminotransferase, aspartate aminotransferase, and alkaline Phosphatase enzymes, as well as hepatic, cardiac, and renal TNF-α and 70-kilodalton heat shock proteins (HSP70)	[46]
24	Renal Disorders	Two-kidney, one-clip procedure using a silver clip (internal diameter 0.25 mm) to induce renovascular hypertension (RVH)	Nine weeks of swimming (Wistar albino rats) after RVH	↓ RVH ↓ Pro-inflammatory cytokines (TNF-α, IL-2, IL-6), lipid peroxidation (malondialdehyde), and neutrophil infiltration (myeloperoxidase activity) ↑ Antioxidant glutathione and catalase levels in the cardiac tissue.	[47]
25	Renal Disorders	Experimental autoimmune encephalomyelitis was induced in 4 weeks	Regular EXE (forced swimming) for 6 weeks	↓ Body mass loss	[48]
26	Liver Disorders	Subcutaneous injection of myelin oligodendrocyte glycoprotein emulsified in CFA. Intraperitoneal injection of pertussis toxin (twice, two days apart)	6-week swimming EXE	↓ Fetuin-A levels ↑ AMP-activated protein kinase and nicotinamide adenine dinucleotide	[49]
27	Liver Disorders	HFD-induced non-alcoholic fatty liver disease	HFD and EXE were provided for 12 consecutive weeks	↓ HFD-induced obesity (weight and fat composition), adipocyte hypertrophy, liver lipid accumulation, pathological steatosis, and nonalcoholic fatty liver disease (NAFLD)	[50]
28	Liver Disorders	HFD (To induce a non-alcoholic fatty liver model in 6 weeks)	Swimming EXE (5 d/wk for 8 wk) + silymarin and vitamin C (supplemental gavage for 8 wk)	↓ Liver inflammatory biomarkers TNF-α and IL-1β ↑ TOC and PPAR-α.	[6]
29	Liver Disorders	N/A	Group 1: sedentary (no EXE). Group 2: two weeks of adaptation (10 min swimming, followed by daily increments of 5 min until reaching 60 min) Group 3: two weeks of adaptation + one intense training session (intense EXE without any previous training). Group 4: two weeks of adaptation + 18 weeks of moderate ST (in which rats swam 60 min every other day, three times a week) + one session of intense swimming EXE.	The training plus strenuous exercise obtained increased levels of secretory immunoglobulin A (sIgA) and polymeric immunoglobulin receptor (pIgR) in the proximal intestine, higher levels of sIgA in the distal segment, and lower mRNA expression of some sIgA- and most pro-inflammatory pIgR-producing cytokines.	[51]
30	Liver Disorders	Standard chow (10% lipid) or high-fat chow (60% lipid) for 22 weeks.	EXE protocol: 10 weeks of swimming, the first two weeks adaptation (6 min/day until 60 min/day, 5 times/week) without an increase in weight in the tail. Eight weeks with 40–60% of VO_2max_	The swimming program, even accompanying the HFD: ↓ Body mass, hyperglycemia, hyperinsulinemia with insulin resistance, hypertrophy of the adipocytes (with inflammatory infiltrate), hypertrophy of the pancreatic islets, dyslipidemia, altered hepatic enzymes and inflammatory cytokines, and NAFLD with alterations in gene expression of hepatic lipogenic and oxidative proteins.	[52]
31	Liver Disorders	Athymic BALB/c nude mice were implanted orthotopically with human liver cancer cells with high metastatic potential	Moderate swimming (8 min/day, 9 weeks) overload swimming (16 and 32 min/day, 9 weeks)	Moderate swimming ↑ DA levels in the prefrontal cortex, serum, and tumor tissue suppressed growth, decreased lung metastasis of transplanted liver cancer, and prolonged survival suppressed the TGF-β1 Overloading swimming had the reverse impact.	[53]
32	Liver Disorders		Young (4 weeks old) and middle-aged (14 months old) animals. 60–90 min of swimming EXE daily, 5 days per week for 9 weeks. Middle-aged (18-month-old) and old (28-month-old) male rats. Four times a week, 60–90 min/day of treadmill exercise for 9 weeks.	Swimming resulted in: ↓ Protein carbonyl Proteasome activity was increased Treadmill running resulted in: ↓ The binding of transcription factor NF-κB to the target DNA ↑ Reduced glutathione in the liver of old rats	[54]
33	Liver Disorders	The normal control group had a normal diet, and the other groups were all fed a 60% kcal fat HFD for 16 weeks.	Chronic and acute swimming EXE training	Both long-term and short-term swimming EXE training ↓Body weight and visceral fat mass, hepatic lipid accumulation Amelioration in insulin resistance (IR) and inflammation. Inhibited PPAR-γ and its target genes expression, containing CD36, severe combined immunodeficiency 1, and perilipin-2	[55]
34	Liver Disorders		The swimming protocol for exercise groups in the first week was as follows: 1st day, 10 min; 2nd day, 20 min; 3rd day, 30 min; 4th day, 40 min; and 5th day, 50 min. In the fructose-enriched diet (FED) group, 20% fructose (*w*/*v*) was mixed into the drinking water of the rats for 16 weeks.	MDA levels and SOD activities were higher in Group FED + EXE. Caspase-3 (Cas-3), receptor activator of nuclear factor kappa-Β ligand, and TNF-α expressions were higher in Group FED, and HSP70 expression was lower.	[56]
35	Liver Disorders	Obesity induced by HFD	Vitamin D and Swimming EXE (30 min, 5 days a week, 6 weeks)	Enhanced HFD-induced weight gain ↓ Hepatic steatosis Developing the serum lipid profile, degree of inflammation, and serum adipokine levels. ↓ Expression of fatty acid transport protein 4 and Toll-like receptor 4 in adipose tissue and the liver	[57]
36	Reproductive System Disorders	HFD-induced obesity	Variable EXE loads (5 days per week, 8 weeks) Gradually elevated training load to 2 h per day (moderate intensity group with obesity) and 2 h twice a day high-intensity group with obesity)	Only the moderate intensity group with obesity showed a decreased overweight-induced OS ↓ Expression of NF-κB and proinflammatory cytokines Reversed the decrease in mRNA and protein expression of testosterone synthases, serum testosterone level, and sperm quality.	[58]
37	Pancreatic disorders	HFD, STZ (30 mg/kg, i.p.) Ovariectomized (OVX)	Swimming EXE (1 h/day, 8 weeks)	↓ Inflammatory cytokines and tissue damage ↑ Sirtuin 1 (SIRT1) by training was associated with ↓ NF-κB-p65 and IL-1β expression and preventing tissue damage	[59]
38	Pancreatic disorders	HFD (4 weeks) and STZ (35 mg/kg, i.p.)	12 weeks of swimming	↓ Expression levels of miR-146, NF- κB and inflammatory cytokines (IL-6, TNF-α, and IL-1β) ↑ Pancreatic expression levels of TNF receptor-associated factor 6 and interleukin-1 receptor-associated kinase 1	[60]
39	Pancreatic disorders		Control group, exercised group (3 weeks of swimming EXE), stressed group (3 weeks of immobilization stress), and stressed group practicing exercise (3 weeks of EXE, concomitant with 21 daily sessions of stress)	Physical EXE: ↑ Pancreatic IL-10 and total antioxidant capacity ↓ Pancreatic TNF-α and malondialdehyde	[61]
40	Pancreatic disorders	HFD and STZ (35 mg/kg, i.p.)	Swimming (60 min/5 days a week) for 10 weeks	↓ Lymphocytes, monocytes ↑ Neutrophils ↓ Levels of CRP, IL-6, and TNF-α	[62]
41	Pancreatic disorders	Obese Zucker diabetic fatty (ZDF) rats were used	Swimming 1 h/day, 3 days/week, for 11 weeks	↓ Hyperuricemia and IL-6 and TNF-α levels, Sustained the weight of the pancreas at near normal and ↓ Expression of TNF-α and IL-6 in the pancreatic islet cells	[63]
42	Diabetes and Related Disorders	HFD for 2 weeks + STZ (35 mg/kg, i.p.)	ST for four weeks	↓ Body weight, glucose, and insulin resistance, ↓ Attenuated memory disorders ↓ IL-6, IL-1β, TNF-α, and glutamate, ↑ BDNF levels in the hippocampus and prefrontal cortex of diabetic mice	[5]
43	Diabetes and Related Disorders	HFD (4 weeks) STZ (35 mg/kg, i.p.)	Swimming (30 min, 3 d/w, 8 weeks) Metformin (MET) (100 mg/kg)	↓ Glucose, homeostatic model assessment of insulin resistance index, low-density lipoprotein, nesfatin-1 levels in the plasma, TNF-α, IL-1β, IL-6, Cas-3, Bcl-2-associated X protein (Bax), and inflammatory response in tissues ↑ Plasma insulin, high-density lipoprotein (HDL), and tissue Bcl-2	[64]
44	Diabetes and Related Disorders	12-week HFD STZ (i.p.)	Swimming: Gradually increased to 60 min/day without weights over 8 weeks. Resistance training: Ladder climbing with load progression from body weight to 100%, 60 min/day. Aerobic exercise: Treadmill running increased to 15 m/min for 60 min/day. HIIT (high-intensity interval training): Treadmill intervals (10 × 4 min high-intensity runs with 2 min rest), speed increased from 16 to 26 m/min.	Exercise training decreased body weight and body fat percentage and caused a decrease in crown-like structures and the expression of inflammatory parameters, mainly in epididymal white adipose tissue (eWAT). HIIT was the most effective in ↓ body fat percentage, ↑muscle mass, and reducing eWAT adipocyte size	[65]
45	Diabetes and Related Disorders	STZ injection (50 mg/kg, i.p.)	4 weeks of swimming	↓ IL-1β, Bax, and Cas-3, and ↑ Bcl-2 ↓ The thickness of the inter-alveolar septum and mean alveolar area in diabetic mice.	[66]
46	Diabetes and Related Disorders	High-sugar and high-fat diet A single of 1% STZ	Non-weight-bearing swimming, 60 min per day, 6 days per week for 8 weeks	In the diabetic exercise group: ↓ Fasting blood glucose levels and insulin resistance index were reduced Myocardial fibrosis was remarkably relieved ↓ mRNA expression of type I and III collagen fibers and TGF-β1 in rat myocardial tissue	[67]
47	Diabetes and Related Disorders	6-week HFD and STZ (i.p.)	Sodium-glucose transport-2 inhibitor dapagliflozin swimming	Enhanced the diabetic-induced histopathological changes in the myocardium ↓ Serum blood glucose, creatine kinase MB (CKMB), lactate dehydrogenase (LDH), myocardial MDA, and mRNA expression of TNF-α, IL-1β, TGF-β, matrix metalloproteinase-9 (MMP-9), and the immune expression of caspase-3 ↑ Serum insulin, myocardial antioxidants glutathione (GSH) and catalase, and increase the immune expression of the microtubule-associated protein 1 light chain 3	[68]
48	Diabetes and Related Disorders	Bilateral ovariectomy, HFD feeding, single dose of STZ	8 weeks of swimming	There was a major difference in the protein expression between the exercise and OVX diabetic groups	[69]
49	Diabetes and Related Disorders	STZ (i.p.)	DIG group (pregnant diabetic rats): insulin 5 U/day, i.p (2 U at 10 am and 3 U at 7 pm) DIEG group (pregnant diabetic rats treated with insulin and subjected to swimming): one dose of 2 U/day at 7 pm	↑ The expression of IL-1β, TNF-α, vascular endothelial growth factor A, and type I collagen, and a higher apoptotic index in the placentas of the DG (pregnant diabetic rats) and DEG (pregnant diabetic rats subjected to swimming) groups But ↓ in glycemia in the latter group	[70]
50	Diabetes and Related Disorders	OVX	Genistein (1 mg/kg, eight weeks; daily subcutaneous [s.c.]) 8 weeks of swimming	↑ miR-132, miR-146b, matrix metalloproteinase-2, NF-κB, extracellular signal-regulated kinases, vascular endothelial growth factor, TNF-α, IL-1β proteins, and MDA factor in the OVX + diabetes + genistein group ↓ GSH The combination of EXE and genistein was more effective than each treatment alone. ↓ Impairment of retinal neovascularization in the OVX diabetic rats	[71]
51	Diabetes and Related Disorders	High-fat diet (60%) for 45 days, then STZ (40 mg/kg body) and nicotinamide (200 mg/kg body) i.p. injection	Swimming EXE (40 min, 5 days per week) or oral melatonin (10 mg/kg bwt per day) alone or in combination.	↓ The development of hyperglycemia, insulin resistance, dyslipidemia, hyperleptinemia, and hypoadiponectinemia levels demonstrates metabolic syndrome. Improves ADB EXE combined with oral melatonin synergistically decreases serum corticosterone and hippocampus tissue level of inflammatory cytokines, and improves ATP level Upregulate the expression of BDNF, peroxisome proliferator-activated receptor gamma coactivator-1α (PGC-1α), and mitochondrial biogenesis-related proteins, glucose transporter type 4 (GLUT4) in hippocampus tissue.	[72]
52	Diabetes and Related Disorders	N-acetylcysteine (50 mg/kg daily, for 21 days, i.p.) anti-CD4/CD8 25 μg/mL on days 0, 7, 14, and 21	Daily 30 min of swimming	↓ Lymphocytes and inflammatory processes, ↑ Relative neutrophil numbers. ↑ Nuclear, cytoplasmic volume, and an intense insulin receptor marking compared to the nucleotide oligomerization domain group without treatment.	[73]
53	Diabetes and Related Disorders	HFD (58% fat) STZ (35 mg/kg, i.p.)	Swimming (5 days per week for 4 weeks)	↓ Anhedonia and depression-like behavior ↓ Glucose and inflammatory cytokines in the serum	[74]
54	Diabetes and Related Disorders		Swimming (60 min/day, 5 days/week for 8 weeks)	↑ Insulin sensitivity, serum IL-4 levels, insulin receptor substrate 1, and protein kinase B phosphorylation	[75]
55	Diabetes and Related Disorders	A 5 × 5 mm fragment of the right uterine horn was sutured to the peritoneum to induce endometriosis.	Light EXE (swimming once a week) Moderate EXE (swimming 3 times a week) Intense EXE (swimming 5 times a week)	↓ Size of endometriotic lesions (greater reduction in moderate and intense activity) ↑ Fasting blood sugar levels ↓ MMP-9 and proliferating cell nuclear antigen levels	[76]
56	Diabetes and Related Disorders	HFD (45 days, 60% fat), followed by i.p. injection of STZ (40 mg/kg) and nicotinamide injection after 15 min	Melatonin supplement (5 mg/kg twice daily) Swimming (40 min/day, 5 days/week)	Ameliorated hypertension, IR, and biochemical alteration induced by diabetes ↑ EXE performance. The expression of GLUT4 mitochondrial biogenesis-associated proteins, such as peroxisome PGC-1α, nuclear respiratory factor, and mitochondrial transcription factor-A, increased in skeletal and cardiac muscle in the diabetes plus melatonin and exercise group.	[77]
57	Diabetes and Related Disorders	HFD (12 weeks)	HIIT	In the liver: ○ Enhances the insulin immunosensitivity in the islets ○ Improves β-oxidation and PPAR-α ○ ↓ Body mass, blood glucose, glucose tolerance, hepatic lipid profile, plasma levels of inflammatory cytokines, adiposity, hepatic steatosis, lipogenesis, and PPAR-γ levels In skeletal muscle: ○ Enhances PPAR-α and GLUT4 ○ ↓PPAR-γ levels	[78]
58	Diabetes and Related Disorders	HFD STZ (35 mg/kg, i.p.)	Swimming (60 min/5 days a week) for 10 weeks	↓ Lymphocytes, monocytes ↑ Neutrophils ↓ Levels of CRP, IL-6, and TNF-α	[62]
59	Diabetes and Related Disorders	HFD	Swimming (1 h/day for 5 days/week for 8 weeks)	In the HFD + Exercise group: less increase in body weight ↑ IL-10 ↓ TNF-α ↓ Cardiac fibrosis ↑ The percentage ejection fraction and fractional shortening	[79]
60	Diabetes and Related Disorders	Genetic model- ZDF rats	Acute EXE: 1 session of swimming until exhaustion Long-term EXE: Initially, rats swam 15 min/d (5 d/wk) gradually increased in 1 week, 1 h/d, 3 d/wk, for 11 wks	Short-term EXE: lower values of glycemia and insulinemia, ↑ inflammatory profile, and OS Long-term training: ameliorating glycaemic and lipidic dysmetabolism, ↓ inflammatory and oxidative markers.	[80]
61	Diabetes and Related Disorders	Genetic model- ZDF rats	Swimming (1 h/day 3 days/week for 12 weeks)	Preventing the hyperglycemic, hyperinsulinemic, and dyslipidemic pattern Without body weight change ↑ Plasma adiponectin ↓ CRP	[81]
62	Cardiovascular Disorders	Isoproterenol (ISO) i.p.	Copper nanoparticle (CuNP) (1 mg/kg/day, orally, 4 weeks) Wortmannin (1 mg/kg/day, i.p.) for 4 weeks Swimming (90 min, 5 days/4 weeks)	CuNP and exercise: ↓ CKMB, cardiac troponin I (cTnI), LDH Improve nitrite/nitrate concentration and lipid profile ↓ OS Wortmannin reversed these changes and prevented the reduction of phosphorylated glycogen synthase kinase-3 beta. Low-dose CuNP and EXE: prevent myocardial infarction	[82]
63	Cardiovascular Disorders	Fed a 0.9% Na^+^ (equivalent to 2% NaCl)	ST (22 weeks)	Normalized BP levels Preserved the renal function ↓ Glomerular shrinkage ↓ CSF [Na^+^] levels	[45]
64	Cardiovascular Disorders	D-galactose injection (i.p.)	ST in warm water 60 min/day, five days/week	↑ SIRT1, PGC-1α, and AMP-activated protein kinase alpha 1 subunit Suppressed aging-associated inflammatory cytokines	[83]
65	Cardiovascular Disorders	RVH surgery	ST (9 weeks)	↑ Immunohistochemical staining of aortic endothelial nitric oxide synthase ↓ Inducible nitric oxide synthase staining Reversed the changes in echocardiographic and oxidative parameters ↓ Oxidative injury	[47]
66	Cardiovascular Disorders	OVX rats	ST (4 weeks) Saline, an estrogen receptor beta agonist (diarylpropionitrile [DPN]), an estrogen receptor alpha agonist (propyl pyrazole triol [PPT]), or oxytocin	ST: ○ Prevented weight gain, ↓ Disorganization of cardiac muscle fibers ST or DPN, PPT, or oxytocin treatments: ○ Suppressed the Increase of TNF-α concentration DPN, PPT, or oxytocin treatments + ST: ○ ↓ IL-6 concentration	[84]
67	Cardiovascular Disorders	HFD (75 days)	Four groups: 1: HL, sedentary, subjected to swimming stress (5 min per day, 5 times per week); 2: NAT submitted to a swimming protocol (1 h per day, 5 times per week) from the 16th day of the experiment; 3: PRO, sedentary, submitted to swimming stress and received oral propolis extract from the 16th day of the experiment; 4: HL + NAT + PRO were swum and received propolis	Swimming and propolis alone and in combination: Prevented the LVH, atherosclerosis, and arterial and ventricular inflammatory responses ↓ CD40 L expression ↑ HDL cholesterol plasmatic levels	[85]
68	Cardiovascular Disorders	mice with chronic Chagas disease	30 min/daily, 5 times a week, 8 weeks	↑ Volume density of capillaries ↓ Volume density of collagen fibers and cross-sectional area of cardiomyocytes	[86]
69	Cardiovascular Disorders	ISO (S.c.)	Flaxseed supplementation (6 weeks) ST	Flaxseed supplementation + ST: ↑ HDL and paraoxonase-1 ↓ Cardiac troponin, Il- 1β, and TNF- α levels Satisfactory level of cTnI, pentraxin 3, Il-1β, and TNF-α	[87]
70	Cardiovascular Disorders		Treadmill running/ST	In both exercising groups: ○ Elevated HDL cholesterol ○ Elevated platelet aggregation, together with higher platelet distribution width and mean platelet volume values. ○ elevated red blood cell (RBC) ○ Higher Plasma and platelet serotonergic system In the swimming group: ○ Higher RBC and hematocrit ○ Decreased mean corpuscular hemoglobin and mean corpuscular hemoglobin concentration value ○ Elevated concerning the plasma and platelet, norepinephrine, and epinephrine concentrations	[88]
71	Cardiovascular Disorders	Surgical ligation of the left coronary artery.	ST (60 min/day, 5 days/week, for 8 weeks)	Enhancement of diastolic function (by lowering the left ventricular end-diastolic pressure) ↑ Lipid peroxidation ↓ IL-10	[89]

**Abbreviations**: 6-hydroxydopamine (6-OHDA); Alzheimer’s disease (AD); anxiety and depression-like behaviors (ADB); β-amyloid peptide (Aβ); B-cell lymphoma-2 (Bcl-2); Bcl-2-associated X protein (Bax); blood pressure (BP); brachial plexus avulsion (BPA); bromodeoxyuridine (BrdU); cardiac troponin I (cTnI); caspase-3 (Cas-3); cerebrospinal fluid (CSF); complete Freund’s adjuvant (CFA); copper nanoparticles (CuNP); creatine kinase MB (CKMB); C-reactive protein (CRP); diarylpropionitrile (DPN); dopamine (DA); electroacupuncture (EA); epididymal white adipose tissue (eWAT); exercise (EXE); fructose-enriched diet (FED); glial fibrillary acidic protein (GFAP); glucose transporter type 4 (GLUT4); glutathione (GSH); glutathione reductase (GR); high-density lipoprotein (HDL); high-intensity interval training (HIIT); insulin resistance (IR); interleukin-1 beta (IL-1β); interleukin-2 (IL-2); interleukin-4 (IL-4); interleukin-6 (IL-6); interleukin-10 (IL-10); intracerebroventricular (ICV); intraperitoneally (i.p.); isoproterenol (ISO); lactate dehydrogenase (LDH); malondialdehyde (MDA); matrix metalloproteinase-9 (MMP-9); matrix metalloproteinase-13 (MMP-13); maximal oxygen uptake (VO_2max_); neuronal nuclei (NeuN); nonalcoholic fatty liver disease (NAFLD); nuclear factor kappa light-chain-enhancer of activated B lymphocytes (NF-κB); oxidative stress (OS); ovariectomized (OVX); peroxisome proliferator-activated receptor alpha (PPAR-α); peroxisome proliferator-activated receptor gamma (PPAR-γ); peroxisome proliferator-activated receptor gamma coactivator-1α (PGC-1α); photobiomodulation therapy (PBMT); polymeric immunoglobulin receptor (pIgR); propyl pyrazole triol (PPT); red blood cell (RBC); renovascular hypertension (RVH); secretory immunoglobulin A (sIgA); streptozotocin (STZ); subcutaneous (s.c.); superoxide dismutase (SOD); swimming training (ST); transforming growth factor beta (TGF-β); total antioxidant capacity (TOC); tumor necrosis factor-alpha (TNF-α); Zucker diabetic fatty (ZDF) ↑: increase; ↓: decrease. **Experimental groups:** DG: pregnant diabetic rats; DEG: pregnant diabetic rats subjected to swimming; DIG: pregnant diabetic rats; DIEG: pregnant diabetic rats treated with insulin and subjected to swimming; HL: sedentary rats subjected to swimming stress (5 min/day, 5 times/week); NAT: rats submitted to a swimming protocol (1 h/day, 5 times/week) from day 16; PRO: Sedentary rats submitted to swimming stress and treated with oral propolis extract from day 16.

Goes et al. (2014) [36] investigated the neuroprotective effects of swimming training (ST) in a mouse model of PD induced by 6-OHDA. Mice underwent a 4-week swimming regimen after 6-OHDA administration. The study found increased falls during motor testing, elevated reactive species and IL-1β, enhanced glutathione reductase (GR) and glutathione S-transferase activities, reduced dopamine levels, inhibited glutathione peroxidase activity, impaired memory, and depressive-like behavior. The researchers concluded that exercise may help mitigate cognitive and motor deficits, depressive symptoms, oxidative stress, and neuroinflammation linked to PD through its antioxidant and anti-inflammatory properties [36].

Kuzmina et al. (2022) [90] conducted an investigation into swimming disorders among individuals diagnosed with PD. The study cohort consisted of forty patients with PD, who were segregated into two groups based on whether swimming was impaired. The findings revealed that 60% of the subjects reported experiencing difficulties with swimming. These individuals demonstrated elevated levels of anxiety compared to those without swimming disorders. Notably, there was no significant association between the occurrence of swimming disorders and the extent of motor symptoms associated with the disease [90].

#### 3.1.3. Neuropathies

Hsieh et al. (2022) [34] studied the effects of cold-water swimming (CWS) on pain and functionality in a rodent model of brachial plexus avulsion (BPA). Rats underwent CWS or sham training twice daily for 5 min over a 2-week period following BPA. The results showed significant improvement in motor and sensory deficits, reduced nerve vacuole formation, and decreased inflammatory cell infiltration in the treatment group. Although BPA increased the expression of substance P and ionized calcium-binding adaptor molecule 1 in dorsal horn neurons, CWS prevented the overexpression. The study concluded that CWS has beneficial effects on functional recovery and pain modulation in the early stages of BPA [34].

Debastiani et al. (2019) [35] investigated the impacts of sericin silk protein and swimming on peripheral nerve repair in Wistar rats. The study protocol consisted of swimming sessions five days a week for three weeks, with a 10% body weight overload and a progressive duration (15, 20, and 25 min per day). The experimental findings demonstrated a substantial improvement in swimming performance among subjects in the injury-swimming group compared to those in the injury, injury-sericin, and injury-sericin-swimming groups. Notably, the latter group had a significantly larger quantity of viable and non-viable nerve fibers with diameters less than 4 μm. Based on these observations, the investigators proposed that the combined application of sericin and swimming exercise may exert a regulatory effect on peripheral nerve repair. This effect is characterized by a marked proliferation of smaller-diameter fibers, suggesting a potential synergistic mechanism in the regenerative process [35].

### 3.2. Rheumatological Disorders

Inflammatory arthritis refers to conditions characterized by joint inflammation, which can potentially lead to cartilage degradation and subsequent degenerative changes. These changes may manifest as functional impairment and joint instability. Among the representative diseases associated with inflammatory arthritis are ankylosing spondylitis (AS), rheumatoid arthritis (RA), and gouty arthritis. Each of these conditions exhibits distinct pathophysiological mechanisms and clinical presentations, yet they share the common feature of inducing inflammatory processes within the affected joints [15]. Ankylosing spondylitis is considered an autoimmune disorder. Osteoarthritis (OA) develops over time due to the aging and wear and tear of the joints. This wear and tear worsens over time.

#### 3.2.1. Osteoarthritis

Swimming is recommended by the Osteoarthritis Research Society (OARSI), the American College of Rheumatology (ACR), and the European League Against Rheumatism (EULAR) as a non-pharmacological approach to managing symptoms of knee osteoarthritis (KOA) [91]. The etiology and pathogenesis of osteoarthritis are associated with inflammatory processes, articular cartilage degradation, and OS within the joint microenvironment. The factors mentioned above demonstrate a complex interrelationship that may be involved in the emergence and continuation of the pathological condition [40]. A study suggests that moderate-intensity mechanical stress, caused by exercise, may alter the signaling pathways of inflammatory mediators implicated in osteoarthritis pathophysiology. These mediators include interferon-gamma (IFN-γ), TNF-α, IL-1β, and IL-6. This modulation potentially influences the regulation of proteoglycan and collagen synthesis, which could have implications for joint degradation processes. It also indicates that exercise may induce an anabolic and protective response via several mechanisms: increased expression of anti-inflammatory cytokines such as IL-10 and interleukin-4 (IL-4) and enhanced secretion of growth factors, notably transforming growth factor beta (TGF-β), resulting in activation of the antioxidant defense system, including glutathione (GSH) and superoxide dismutase (SOD) [40].

Swimming is often considered a beneficial activity for those with OA due to its characteristic of having a low impact. However, current scientific evidence of positive effects on vascular disorders and inflammation in patients with OA is limited, particularly in contrast to activities like cycling, which are land-based. While swimming may offer particular advantages, the comparative effects of swimming versus land-based exercises on the function of the vascular system and markers relating to inflammatory responses in OA patients remain insufficiently studied. Further investigations are necessary to establish the relative merits of these modalities in managing the vascular and inflammatory aspects of OA. This study underscores the significance of OA research in humans, contrasting with the prevalence of animal models in similar disease studies [92].

In another investigation in 2024, Zhu et al. [37] investigated the effects of swimming exercise on articular cartilage in old mice that were affected with patellar osteoarthritis. Using 3-month-old (young) and 18-month-old (aged) groups, as well as an 18-month-old group with exercise (swimming), the researchers found that aged mice showed decreased stride length, reduced chondrocyte count, lower type II collagen and aggrecan expression, increased inflammatory cell counts, and elevated modified Mankin’s scores compared to young mice. Diversely, the swimming intervention group showed improvements in these parameters. This group demonstrated smoother articular surfaces, as observed through histological analysis, with normal chondrocyte appearance and reduced proteoglycan loss. The authors concluded that swimming exercise can reduce blood inflammatory cells, improve chondrocytes and matrix composition, and preserve cartilage morphology in aged mice with knee osteoarthritis, suggesting a potential protective effect of swimming (Table 2) [37].

A 2023 study by Saber et al. [38] examined the potential additive impacts of curcumin and swimming on osteoarthritis progression in a rat model. The experimental protocol comprised daily 20-min swimming sessions combined with oral curcumin administration (200 mg/kg) over a 4-week period. The researchers observed a reduction in pain and joint stiffness, improvement in knee joint features, a decrease in serum C-reactive protein (CRP) and cartilage oligomeric matrix protein levels, and alterations in miR-130a and histone deacetylase 3 (HDAC3) expression after swimming. The combined therapy also upregulated peroxisome proliferator-activated receptor gamma (PPAR-γ), downregulated NF-κB and its inflammatory targets, such as TNF-α and IL-1β, and reduced the expression of matrix metalloproteinase-1 and matrix metalloproteinase-13 (MMP-13). The authors suggested that the therapeutic effects of combining curcumin with swimming in osteoarthritis may be mediated through the miR-130a/HDAC3/PPAR-γ signaling axis [38].

Martins et al. (2022) [39] evaluated the outcome of electroacupuncture (EA), swimming, and their combined application on a rodent model of osteoarthritis (OA). The research utilized a rat model of talar monoarthritic, with subjects allocated into three treatment groups seven days following OA induction. Nociceptive behavior was evaluated using cold stimulation (using acetone, cold allodynia), Von Frey filaments (tactile allodynia), and Paw Flick Immersion tests. Results indicated that the EA group exhibited reduced nociceptive marks in the cold stimulation test, increased latency time in thermal cold and heat hyperalgesia assessments, and decreased *N*-acetyl-β-D-glucosaminidase levels. Also, the swimming group demonstrated reduced edema without increasing inflammatory infiltrates or congestion, as evidenced by histological assessment and TNF-α levels. The combined EA + swimming treatment decreased the levels of neutrophils, monocytes, myeloperoxidase (MPO), and glutamate in the cerebrospinal fluid. Interestingly, while both individual treatments showed benefits, the combination of EA + swimming did not demonstrate superior effects on nociceptive behavior compared to the isolated protocols. These findings suggest that, while both EA and swimming may offer distinct benefits in managing OA symptoms, their combination may not necessarily provide additive effects in all aspects of the condition, particularly in pain perception [39].

In a 2023 study, da Silva, L. A. et al. [40] examined the efficacy of a treadmill in association with swimming in an animal model with osteoarthritis using four groups of 12 male Wistar rats. The treadmill protocol involved a progressive increase in duration and speed over six weeks, with intensities ranging from 50% to 60% of maximal oxygen uptake (VO_2max_), while the swimming protocol consisted of a one-week adaptation period followed by six weeks of 20 min daily sessions in 32 °C water on alternating weekdays. Results indicated that treadmill exercise was more effective in reducing pro-inflammatory cytokines (TNF-α, IFN-γ, IL-6, and IL-1β), while positively influencing anti-inflammatory factors (TGF-β, IL-4, and IL-10), improving the balance between oxidation and reduction, and preserving chondrocyte numbers, as evidenced by histological evaluation. These findings suggest that while both forms of exercise may offer benefits in osteoarthritis management, treadmill exercise may provide superior outcomes in terms of inflammatory modulation and joint tissue preservation in this animal model [40].

#### 3.2.2. Rheumatoid Arthritis (RA)

RA is a systemic chronic autoimmune disorder. It is widely recognized that concurrent inflammation of the synovial tissue in multiple joints can lead to joint deterioration [7]. The pathophysiology of RA is primarily mediated by activated lymphocytes and macrophages (MΦ) [42]. Individuals with rheumatic conditions have an elevated risk of developing diabetes mellitus. Conversely, patients with diabetes who subsequently develop rheumatic disorders may experience inconsistent blood glucose levels [93]. Pharmacological interventions employed in the management of rheumatic diseases, including corticosteroids, calcineurin inhibitors, and hydroxychloroquine, present significant risks for diabetes. Research suggests that physical activity may confer benefits in RA through its impact on the immune system [42].

In 2024, Goto et al. [7] investigated the role of frequent activity on cognitive status and protein carbonyls in aged rat brains. The study utilized young (4 weeks old) and middle-aged (14 months old) Wistar rats, who underwent 9 weeks of strength training. Results showed improved cognitive functions and decreased brain protein carbonyls in both groups, along with upregulated proteasome activity in skeletal muscle. Middle-aged rats exhibited higher levels of inhibitor of kappa B (I-κB) in the cytoplasm and increased NF-κB, suggesting age-related NF-κB activation through enhanced I-κB degradation. While glutathione reductase protein quantity remained consistent across age groups, eight weeks of exercise reversed the decrease in liver GR activity. Exercised older animals also showed higher serum glucocorticoid levels. The researchers concluded that frequent physical activity may prevent or reverse the changes caused by aging-promoting inflammatory processes, based on the observed effects on GR activity and the anti-inflammatory properties of glucocorticoids [7].

Siqueira et al. (2017) [94] conducted a 16-week randomized controlled trial to evaluate the efficacy of swimming interventions in female patients with RA (Table 3). The study initially included 133 women with RA, of whom 82 completed the full protocol. Participants engaged in a thrice-weekly exercise regimen for 16 weeks, consisting of a 5 min warm-up followed by 15–30 min of lower limb exercises of progressively increasing intensity. The researchers observed significant improvements in disease condition and functional ability in the water-based exercise group, swimming, at both 8 and 16 weeks. They concluded that swimming substantially reduces disease severity, controls the pain, and increases functional capacity in women with RA. This study’s focus on human patients underscores its importance (in comparison with animal studies) and the necessity for further research in this area [94].

Navarro et al. (2010) [42] studied how physical activity affects the function and metabolism of immune cells in experimental arthritis. Female Wistar rats with collagen-induced arthritis (CIA) were exposed to a structured swimming protocol consisting of six weeks of exercise sessions lasting 60 min, five days/week. The researchers observed increased lymphocyte proliferation and macrophage production of H_2_O_2_. Exercise training prevented CIA-induced immune cell activation and elevated the amount of corticosterone in plasma, progesterone, and IL-2. The study concluded that chronic exercise can counteract the impact of CIA on the immune system, suggesting a potential therapeutic role for exercise in managing arthritis-related immune dysfunction [42].

#### 3.2.3. Gout

Gout is a long-term inflammatory disorder mainly recognizable by joint alterations that cause acute pain. The condition is associated with hyperuricemia, an excessive accumulation of uric acid in the body due to disrupted purine metabolism. This biochemical imbalance leads to recurrent episodes of paroxysmal joint inflammation [15]. Excessive muscular exertion increases uric acid concentrations, activating the nucleotide-binding oligomerization domain-like receptor. This family of receptors includes the pyrin domain-containing NLR family pyrin domain-containing 3 (NLRP3) inflammasome. Activation of the NLRP3 inflammasome induces the secretion of pro-inflammatory cytokines, primarily IL-1β [15]. Elevated uric acid levels contribute to the development of mechanical hyperalgesia. Xanthine oxidase inhibitors may exhibit potential efficacy in alleviating pain associated with over-exercised muscles [15].

Shi et al. (2012) [43] examined the effect of cold-water swimming on a gouty arthritis model mimicking clinical pathogenesis. The researchers induced persistent hyperuricemia in rats by administering oxonic acid and hypoxanthine. During model preparation, the experimental group swam in cold water (10–12 °C) for 10 min daily over 12 consecutive days. Results showed elevated uric acid concentrations in joint cavities across all treatments, with the hyperuricemia with cold water swimming group exhibiting significantly higher levels. This group also demonstrated ankle injuries characteristic of gouty arthritis, including hemangiectasis, congestion, and inflammatory cell infiltration in the synovium and surrounding soft tissue, which were absent in other groups. The study concluded that cold water swimming can induce swelling reminiscent of gouty arthritis in rats with persistent hyperuricemia, suggesting a potential environmental trigger for the condition [43].

### 3.3. Renal Disorders

**Figure 2:**
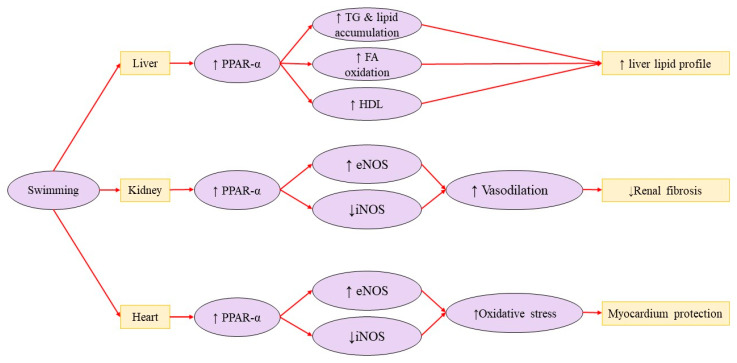
The molecular mechanism of the swimming effect on crucial organs, such as the liver, kidney, and heart, involves regulating PPAR-α. Regular swimming activates PPAR-α expression in the liver, kidney, and heart, leading to multiple anti-inflammatory and antioxidant effects. In the liver, PPAR-α upregulation enhances fatty acid oxidation, increases HDL, and decreases triglyceride (TG) and lipid accumulation, improving the overall lipid profile. In the kidney, activation of PPAR-α increases eNOS and decreases iNOS, promoting vasodilation and reducing renal fibrosis. In the heart, swimming-induced PPAR-α activation enhances eNOS signaling and suppresses iNOS, reducing oxidative stress and providing myocardial protection. Altogether, swimming regulates lipid metabolism, improves vascular homeostasis, and mitigates inflammation and oxidative damage through the PPAR-α/eNOS/iNOS axis, contributing to systemic metabolic health. Abbreviations: Peroxisome proliferator-activated receptor alpha (PPAR-α); endothelial nitric oxide synthase (eNOS); inducible nitric oxide synthase (iNOS); fatty acid (FA); triglyceride (TG); high-density lipoprotein (HDL); ↑: increase; ↓: decrease.

Zhao et al. (2023) [44] evaluated the effects of a 12-week moderate swimming regimen on renal function in aged rats. The study found that swimming improved renal function by reducing plasma creatinine, blood urea nitrogen, renal injuries, fibrosis, oxidative stress, and triglyceride levels while also lowering mRNA expression of fibrosis-related genes. Oxidative stress reduction was indicated by decreased malondialdehyde (MDA) levels and increased manganese superoxide dismutase activity. Additionally, the regimen inhibited NF-κB activity and reduced the expression of pro-inflammatory cytokines (monocyte chemoattractant protein-1 [MCP-1], IL-1β, and IL-6) in the kidneys. These benefits were partly attributed to the activation of peroxisome proliferator-activated receptor alpha (PPAR-α), which suggests a mechanism for improved kidney health in aging (Figure 2) [44].

de Souza et al. (2021) [45] studied the potential effects of swimming training (ST) on rats maintained on a high-sodium (HS) diet from weaning. Their findings indicated that ST reduced blood pressure (BP) in HS rats, correlating with a reduction in cerebrospinal fluid (CSF) sodium concentration, attenuation of sympathetically mediated vasomotor tone, and decreased kidney fibrosis. Notably, sedentary HS rats had higher CSF sodium levels, which improved following ST. The researchers concluded that the exercise regimen effectively normalized BP in HS rats in a separate manner from its effects on the balance of water and electrolytes. They hypothesized that this anti-hypertensive effect may involve mechanisms caused by neurogenic factors regulated by CSF sodium levels and renoprotective effects. These results propose that the BP-lowering impact of ST in rats on a high-sodium diet may be more closely associated with alterations in CSF sodium levels and neurogenic mechanisms rather than changes in blood volume [45].

Farzanegi et al. (2020) [46] investigated the effects of ST on doxorubicin-induced toxicity in rat hepatic, cardiac, and renal tissues. The study found that ST reduced malondialdehyde levels and increased antioxidant enzyme activities, especially when combined with garlic extract. Doxorubicin raised serum alanine aminotransferase, aspartate aminotransferase, and alkaline phosphatase levels and increased TNF-α and heat shock protein 70 in liver, heart, and kidney tissues. The researchers concluded that ST, with or without garlic extract, provided protection against doxorubicin-induced toxicity, likely through enhanced antioxidant capacity and reduced OS and inflammatory responses [46].

Kumral et al. (2016) [47] studied the effects of regular exercise on OS and cardiac dysfunction caused by renovascular hypertension (RVH) in Wistar albino rats. RVH in sedentary rats led to increased pro-inflammatory cytokines (TNF-α, IL-2, IL-6), higher lipid peroxidation, and more significant neutrophil infiltration while reducing antioxidant levels in cardiac tissue. A nine-week swimming regimen after RVH induction increased aortic endothelial nitric oxide synthase (eNOS) immunostaining, decreased inducible nitric oxide synthase (iNOS) immunostaining, and reversed changes in echocardiographic and oxidative parameters. The researchers concluded that regular exercise after RVH surgery reduced oxidative injury by balancing the oxidant–antioxidant system, likely through the eNOS system (Figure 2) [47].

Bernardes et al. (2016) [48] investigated the role of frequent activity on metabolic changes and weight loss in female C57BL/6 mice during the inflammatory peak of experimental autoimmune encephalomyelitis (EAE). The study design involved inducing EAE in half of the subjects over a four-week period, followed by a 10-day interval before initiating a six-week forced swimming exercise regimen. Results showed that exercised EAE animals exhibited significantly lower clinical scores and attenuated body mass loss compared to their non-exercised counterparts. However, the researchers concluded that these notable improvements were insufficient to fundamentally alter the metabolic results related to the inflammatory response at the EAE peak. This suggests that while regular exercise may offer some benefits in managing EAE symptoms, its impact on underlying metabolic changes during peak inflammation may be limited [48].

### 3.4. Liver Disorders

Evidence indicates that swimming exercise mitigates hepatic inflammation and lipid accumulation in liver disorders. Huang et al. (2022) [50] explored exercise in the background of non-alcoholic fatty liver disease (NAFLD) through a lipidomic perspective, with a focus on the modulation of lipid and metabolic processes. In a study using C57BL/6J wild-type and apolipoprotein E (ApoE) knockout mice, the animals were assigned to four intervention groups: wild-type mice on a normal chow diet, ApoE knockout mice on a normal diet, ApoE knockout mice on a high-fat diet (HFD), and ApoE knockout mice on HFD with swimming exercise. The outcomes revealed that exercise intervention attenuated HFD-induced obesity, characterized by reduced weight gain, adipogenesis, adipocyte hypertrophy, hepatic lipid deposition, and pathological steatosis. The authors proposed that the 12 weeks of frequent activity intervention alleviated NAFLD by modifying the plasma lipid profile. This study may provide insight into lipid-related pathways involved in exercise intervention-mediated hepatic pathogenesis [50].

Aghaei et al. (2023) [6] investigated the combined influence of swimming and supplementation with vitamin C and silymarin on inflammatory responses in the kidneys, renal histopathology, and OS in elderly male Wistar rats with HFD-induced liver damage. The study utilized 40 rats and employed a 6-week HFD regimen to induce a non-alcoholic fatty liver model. Subsequently, rats in the supplement groups received daily supplemental gavage concurrent with the HFD for 8 weeks, while the exercise groups underwent ST five days a week during a similar time interval. The findings indicated that the co-occurrence of ST and the utilization of silymarin and vitamin C remarkably reduced inflammatory biomarkers of the liver (IL-1β and TNF-α) and increased total PPAR-α and antioxidant capacities. The researchers stated that the combination of exercise, vitamin C, and silymarin supplements demonstrated efficacy in mitigating oxidative stress, hepatic inflammatory response, fat accumulation, and modulating liver enzymes [6].

Godínez-Victoria et al. (2012) [51] examined the intestinal effect of moderate physical activity levels followed by strenuous swimming activity on the release of Immunoglobulin A (IgA) in mice. The researchers divided mice into two groups: one subjected to strenuous activity with prior training and another without previous training. Their findings revealed that the combination of training and strenuous exercise increased IgA secretion in the proximal intestine, potentially due to hepatobiliary contribution. Additionally, they observed a higher concentration of secretory immunoglobulin A (sIgA) in the distal segment of the intestine. The study also noted a decrease in the expression of mRNA related to some IgA-producing cytokines and most cytokines involved with the production of polymeric immunoglobulin receptor (pIgR) in the trained group that underwent strenuous exercise [51].

Schultz et al. (2012) [52] examined the effects of ST on NAFLD and related comorbidities in male C57BL/6 mice over 22 weeks, focusing on fatty acid synthesis in the liver and PPAR-α. In the last 10 weeks, half of the sedentary mice began progressive ST. Results showed that the sedentary high-fat diet group had remarkably higher body mass, adipocyte hypertrophy, hyperglycemia, insulin resistance (IR), inflammation, pancreatic islet hypertrophy, dyslipidemia, and NAFLD, along with changes in liver gene expression, compared to the sedentary low-fat diet group. Swimming training, even with an HFD, reduced weight gain and mitigated other negative outcomes, particularly NAFLD, in both exercised receiving groups. The researchers concluded that ST could reduce the harmful effects of an HFD and sedentary lifestyle, suggesting it is a potential nonpharmacologic intervention for NAFLD, obesity, and IR [52].

Zhang et al. (2016) [53] investigated the impact of swimming on the transplantation of hepatocellular carcinoma in C57BL/6 mice, as well as the neuromodulatory mechanisms involved. Moderate-intensity daily swimming (8 min) for 9 weeks was found to increase dopamine levels, particularly in the mice’s prefrontal cortex, serum, and tumor tissue, leading to a lower tumor burden, fewer metastatic lung nodules, and increased survival. On the other hand, high-intensity swimming has a polarizing impact similar to that of the former. Disruption of these pathways was found to be mediated via the intracellular dopamine type 2 receptor (D2R), which blocked the phosphorylation of extracellular signal-regulated kinase (ERK) and TGF-β1, thereby inhibiting the expression of the epithelial–mesenchymal transition (EMT)-promoting signaling pathway mediated by TGF-β1. The results suggest that moderate exercise may prevent hepatic carcinoma by activating DR2; excessive exercise, however, could worsen the condition. The study has important implications for the role of dopaminergic systems in liver cancer progression and the variable effects of exercise intensity on cancer endpoints, making strides toward a better understanding of how physical activity mediates neuroendocrine function and tumor biology [53].

Goto and Radák (2007) [54] examined the role of frequent swimming and running on hepatic oxidative stress and the brains of middle-aged and elderly rats. Four-week-old and 14-month-old rats swam for 60–90 min, 5 times a week, for a period of 9 weeks. Middle-aged (18 months) and old (28 months) male rats ran on a treadmill 60–90 min daily, 4 times a week, for 9 weeks. Swimming training reduces carbonylated protein and elevated proteasome activity in the brain, improving cognitive function. Treadmill running in old rats decreased NF-κB binding to target DNA and increased glutathione in the liver, suggesting reduced inflammatory reactions due to lower OS. The researchers concluded that moderate exercise is positively impactful in contrast to severe physical activity, which may be damaging, proposing a mechanism similar to hormesis involving ROS as a potential explanation for the anti-aging impact and reduced chance of diseases caused by the elevation of age associated with regular exercise [54].

Zhang et al. (2023) [55] investigated the effects of swimming on IR and NAFLD in mice on an HFD. They found that both short-term and long-term swimming reduced body weight, visceral fat, and liver lipid accumulation while improving insulin sensitivity and reducing inflammation. Molecular analysis revealed that swimming inhibited PPAR-γ expression and its target genes, including CD36, severe combined immunodeficiency 1 (SCD1), and perilipin-2 (PLIN2), as well as modulated factors that regulate PPAR-γ activity. The researchers concluded that swimming enhances lipid metabolism in IR and NAFLD, likely through the PPAR-γ pathway, suggesting its potential benefits for metabolic dysfunctions caused by HFDs [55].

Altintas et al. (2022) [56] investigated the impact of swimming on restoring hepatic damage resulting from a fructose-enriched diet (FED) in Wistar rats. The study employed a progressive swimming protocol over 5 days, with the sessions starting at 10 min and increasing in duration to 50 min. The FED group received 20% fructose (*w*/*v*) through drinking water for 16 weeks. Results demonstrated the FED plus exercise group had considerably higher MDA levels and SOD activities. Caspase-3, receptor activator of nuclear factor kappa-Β ligand (RANKL), and TNF-α expressions were higher in the FED group, while 70-kilodalton heat shock protein (HSP70) expression was decreased relative to other groups. The researchers concluded that a diet enriched in fructose volume elevates oxidative damage, degeneration, inflammatory responses, and hepatic necrosis while increasing TNF-α, RANKL, and Cas expressions and reducing HSP70 expression. Importantly, the study found that swimming exercise largely mitigated these detrimental effects, suggesting its potential as an intervention for fructose-induced liver damage [56].

Kolieb et al. (2022) [57] assessed whether vitamin D and swimming could prevent overweight in male albino rats receiving an HFD, focusing on the expression of Toll-like receptor-4 (TLR-4) and fatty acid transport protein-4 (FATP-4) expression in adipose and hepatic tissue. The study involved 30 rats and a 6-week swimming protocol, starting with 15 min of daily swimming and increasing to 30 min, five days a week. Results showed that both vitamin D supplementation and physical activity mitigated weight gain and fatty liver caused by increased dietary fat, improved serum lipid profiles, reduced inflammation, and positively affected adipokine concentrations in the blood. TLR-4 and FATP-4 expression were downregulated in both hepatic and adipose tissue. Notably, the group receiving both vitamin D and exercise showed improved histopathological outcomes. The researchers concluded that physical activity and vitamin D could counter overweight and HFD-induced hepatic steatosis by lowering TLR-4 and FATP-4, as well as modulating the inflammatory response [57].

### 3.5. Reproductive System Disorders

Yi et al. (2020) [58] examined the impact of different levels of swimming intensity on testicular OS and reproductive function in obese male mice. The research demonstrated that moderate-intensity exercise (2 h daily) effectively mitigated obesity-induced OS, reduced NF-κB expression and proinflammatory cytokines, and restored testosterone synthase expression, serum testosterone concentrations, and parameters related to sperm quality. In contrast, high-intensity exercise (2 h twice daily) did not yield comparable beneficial outcomes. The investigators concluded that obesity contributes to testicular OS and inflammation, subsequently dampening testosterone production and diminishing the quality of sperm. Moderate-intensity activity was found to attenuate these adverse effects through the reduction of testicular OS and inflammation. In contrast, high-intensity exercise demonstrated limited efficacy in improving male reproductive function in obese mice [58].

### 3.6. Pancreatic Disorders

Habibi et al. (2022) [59] investigated the impact of swimming on IL-1β, SIRT1, NF-κB, and histological pancreas damage in an ovariectomized rat model of diabetes. The study utilized 40 female Wistar rats, allocated into sham and ovariectomized (OVX) groups, with OVX rats, further subdivided into OVX, OVX diabetic, and OVX diabetic plus exercise cohorts. The exercise regimen consisted of 1 h of daily swimming for eight weeks, concurrent with a high-fat diet. The findings indicated that swimming considerably reduced inflammatory cytokine levels and tissue damage, correlating with increased SIRT1 expression and decreased expression of IL-1β and NF-κB-p65. The OVX diabetic group exhibited elevated NF-κB-p65 and IL-1β protein levels, along with decreased SIRT1 expression, compared to the sham group. These effects were significantly attenuated by swimming training. The investigators concluded that swimming attenuates inflammatory response in post-menopausal diabetes and may contribute to the prevention of pancreatic dysfunction following menopause by mitigating β-cell inflammation and supporting insulin secretion under estrogen-deficient conditions [59].

Alipour et al. (2020) [60] studied the effects of swimming exercise on inflammatory markers in the pancreas of male Wistar rats with type 2 diabetes (T2D). Diabetes was induced in 28 rats via an HFD and STZ injection, followed by a 12-week swimming regimen for the exercise groups. The diabetic rats exhibited increased levels of miR-146, NF-κB, and pro-inflammatory cytokines (IL-6, TNF-α, and IL-1β), as well as decreased expression of TNF receptor-associated factor 6 (TRAF6) and interleukin-1 receptor-associated kinase 1 (IRAK1), compared to non-diabetic controls. Swimming significantly reduced miR-146a, NF-κB, and cytokine levels while increasing TRAF6 and IRAK1 expression in the exercised diabetic group. The study suggests that swimming may reduce pancreatic inflammation in T2D by modulating key inflammatory markers, supporting the therapeutic potential of exercise in managing diabetes-related complications [60].

Elbassuoni et al. (2019) [61] investigated the effects of long-term physical activity on stress-induced pancreatic changes in adult male albino rats. The study, which used 40 rats divided into four groups, found that prolonged immobilization stress led to hyperglycemia, hyperinsulinemia, an elevated homeostatic model assessment of insulin resistance index (HOMA-IR), and increased exocrine pancreatic injury markers, all of which are associated with oxidative stress and inflammation in pancreatic tissue. Stress negatively impacted pancreatic morphology, but physical exercise demonstrated protective effects through anti-inflammatory and antioxidative mechanisms. This was evidenced by increased pancreatic IL-10 and total antioxidant capacity, decreased TNF-α and malondialdehyde, and amelioration of stress-induced histological changes. The researchers concluded that physical activity significantly mitigates long-term stress-induced pancreatic alterations, highlighting the potential therapeutic benefits of exercise in addressing stress-related pancreatic dysfunction [61].

In 2016, Ghiasi et al. [62] studied the effects of regular swimming exercise on serum CRP, IL-6, and TNF-α in HFD diabetic male Wistar rats. The study utilized 40 rats, with exercise groups undergoing swimming sessions (60 min, 5 days/week) for 10 weeks after administering HFD and STZ injection (35 mg/kg, intraperitoneal) to induce diabetes. The results demonstrated significant increases in monocytes and lymphocytes and the opposite trend for neutrophils, which were countered by the swimming intervention. The diabetic group exhibited elevated levels of CRP, IL-6, and TNF-α, which were remarkably lowered by swimming. Histopathological analysis indicated that swimming may mitigate diabetes-induced damage. The researchers concluded that swimming correlates with improvements in inflammatory responses, inflammatory mediators, and damage in the pancreas in this animal model of diabetes [62].

In 2009, Teixeira de Lemos et al. [63] evaluated the effects of swimming on IL-6, TNF-α, and uric acid in a rat model susceptible to developing type 2 diabetes mellitus (T2DM) (Zucker diabetic fatty [ZDF] rats). The study included 16 obese ZDF (Gmi *fa*/*fa*) rats (8 weeks old, 228.40 ± 4.05 g) and 16 lean ZDF (Gmi^+/+^) rats 8 weeks old, 199.00 ± 3.50 g). The exercise protocol began with 15 min of swimming each day (5 days/week) in 36 °C water, gradually increasing by 15 min daily until reaching 1 h/day for one 1 week. Subsequently, rats swam for 1 h per day, 3 days per week, for an additional 11 weeks. Rats were euthanized 48 h after the final activity time. In diabetic ZDF (*fa*/*fa*) rats, exercise was associated with reduced hyperuricemia and decreased levels of TNF-α and IL-6. Additionally, exercise appeared to maintain near-normal pancreatic weight and IL-6 and TNF-α expression in pancreatic islet cells. The researchers indicated that aerobic activity suppressed inflammation in this animal model [63].

### 3.7. Respiratory Disorders

Zaccarin et al. (2022) [95] conducted a cross-sectional study examining the prevalence of respiratory symptoms and their relationship to training elements among young Italian professional swimmers (*n* = 396). Data were collected via a questionnaire regarding training parameters and respiratory symptoms in 2021 (during summer and winter in the course of training seasons). Analysis of the winter cohort (*n* = 197) revealed considerable associations between training session duration and the incidence of nasal congestion/rhinorrhea and cough. Total weekly training volume was significantly correlated with nasal congestion/rhinorrhea. In contrast, the summer cohort (*n* = 199) demonstrated no statistically significant associations between these variables and respiratory symptoms. Logistic regression models indicated that allergy status significantly predicted nasal congestion/rhinorrhea across both seasons, while asthma status significantly increased the likelihood of cough. The training environment (indoor versus outdoor facilities) did not emerge as a significant factor influencing the studied respiratory symptoms in either season (Table 3) [95].

Bougault et al. (2012) [96] assessed the effects of intensive swimming training on airway morphology and function in elite athletes (Table 3). This research was prompted by the observed high incidence of airway disorders among regular users of chlorinated pools. The study population consisted of 23 elite swimmers during their off-season, 10 individuals with mild asthma, and 10 healthy, non-allergic controls. Histological and immunohistochemical analyses revealed that swimmers exhibited significantly higher numbers of eosinophils and mast cells in the mucosal lining of airways, enhanced submucosal expression of type I and III collagen, and elevated tenascin deposition compared to control groups. Furthermore, swimmers demonstrated more hyperplasia in goblet cells and elevated mucin expression relative to healthy and asthmatic subjects. These findings suggest a potential correlation between prolonged, high-intensity training in indoor chlorinated swimming environments and significant airway remodeling [96].

Font-Ribera et al. (2010) [97] investigated the acute respiratory effects of indoor chlorinated swimming pools, focusing on disinfection by-products (DBPs). The study involved 48 healthy, non-smoking adults who underwent pulmonary function tests and biomarker analyses for airway inflammation, oxidative stress, and pulmonary permeability before and after a 40 min swim (Table 3). Results showed a modest but significant increase in serum Clara cell protein (CC16) levels, a marker of lung epithelium permeability, from 6.01 to 6.21 μg/L, attributed to both physical exertion and DBP exposure, regardless of atopic status or CC16 genotype. Fractional exhaled nitric oxide (FeNO) slightly decreased in atopic individuals, but no significant changes were noted in pulmonary function, surfactant protein D (SP-D), 8-isoprostane, cytokines, or vascular endothelial growth factor (VEGF). The study concluded that the CC16 increase was linked to physical activity and DBP exposure, with no evidence of inflammatory mechanisms involved [97].

Pachalski et al. (1980) [98] assessed the impact of a swimming training plan on paraplegics’ cardio-respiratory systems (Table 3). The study involved 60 participants (34 males, 26 females) over three years. Researchers employed Skibinski’s cardio-respiratory index (SCRI) to assess cardio-respiratory efficiency. Results showed that following the three-year ST program, participants’ SCRI values quadrupled [98]. Numerous human studies on respiratory diseases and the effect of swimming on them have demonstrated a wide range of valuable data for clinical use and the treatment of potential problems.

### 3.8. Diabetes and Related Disorders

Obesity is associated with a long-term, systemic inflammatory response, IR, β-cell dysfunction, and an elevated vulnerability to T2DM. These conditions are implicated in the pathogenesis of various comorbidities, including NAFLD, as well as retinal, renal, cardiac, and vascular damage associated with diabetes. Evidence suggests potential links between obesity-related metabolic dysfunction and other issues such as AD, polycystic ovary syndrome, gout, and AS. Long-term tissue inflammation has been proven to be a significant characteristic of obesity and T2DM, with inflammatory processes observed in insulin-responsive tissues, including pancreatic islets, adipose tissue, hepatic tissue, and skeletal muscle [101].

Shekarchian et al. (2023) [5] evaluated the effects of swimming on cognitive function in type 2 diabetic mice. The study involved diabetic and non-diabetic mice undergoing a four-week swimming training protocol. Diabetic mice showed increased body mass, hyperglycemia, IR, and cognitive deficits in working, spatial, and recognition memory. Biochemical analysis revealed elevated levels of TNF-α, IL-6, IL-1β, and glutamate, accompanied by reduced hippocampal expression of BDNF. Swimming training was associated with reduced body mass, glycemia, and IR, along with improved memory, decreased pro-inflammatory cytokines and glutamate, and increased hippocampal and cortical BDNF expression. The study suggests that swimming training may help restore metabolic balance, reduce neuroinflammation, and enhance neurotrophic signaling, potentially improving cognitive function in T2D [5].

Saki et al. (2023) [100] examined the effects of high-intensity interval training (HIIT), running, swimming, and resistance training on heart rate variability (HRV) and biochemical markers in adolescents with type 1 diabetes (T1D) and their homeostasis. There were 24 subjects with type 1 diabetes (T1D) and 12 normal controls, randomly assigned into three groups: diabetes control (DC), healthy control (HC), and diabetes-training group (DT). Over 12 weeks, participants attended three weekly exercise sessions. Baseline data revealed significant differences in HRV (active vs. T1D group), glycated hemoglobin (HbA1c), fasting blood glucose (FBG), peak oxygen uptake VO_2_ peak amplitude, norepinephrine, and high-density lipoprotein (HDL) cholesterol between the T1D group and healthy controls. The HRV and VO_2max_ were increased, and VO_2_ peak and HDL cholesterol levels were increased after exercise, but HbA1c, NE, and FBG decreased significantly post-intervention. High-intensity interval training might improve cardiac autonomic function, lipid profiles, and catecholamine levels, and improve cardiorespiratory fitness in T1D patients [100].

Özüdoğru et al. (2023) [64] investigated the cardioprotective effects of swimming exercises and metformin on HFD-induced cardiac and aortic changes in a rat model of T2DM, focusing on the Bcl-2/Bcl-2-associated X protein (Bax) signaling pathway. The study involved 27 male Wistar albino rats with T2DM, divided into four groups: HFD, metformin (MET), exercise (EXE), and MET plus EXE. The EXE and MET plus EXE groups participated in 30 min swimming sessions thrice weekly for eight weeks. The HFD group showed elevated glucose, IR, and LDL levels, while HDL showed the opposite trend. The intervention groups experienced reductions in glucose, HOMA-IR, LDL, IL-1β, TNF-α, IL-6, Cas-3, Bax, and histopathological inflammation. These groups also had increased insulin, HDL, and Bcl-2 levels, suggesting that both MET and EXE mitigate cellular damage in T2DM [64].

Guo et al. (2023) [65] examined adipose tissue responses to distinct exercise types in a diabetic mouse model. The study involved an HFD injection of STZ, followed by 8-week pre-exercise interventions (swimming/Resistance training/AERO/HIIT), which served as the treatments. The data revealed an exercise-induced decrease in body weight, fat percentage, inflammation, and crown-like structures, primarily in epididymal white adipose tissue (eWAT). Tyrosine hydroxylase expression led to browning, which happened only in the subcutaneous white adipose tissue (sWAT). HIIT demonstrated the most significant effects, reducing body fat, increasing muscle mass, decreasing eWAT adipocyte size, and elevating oxidative phosphorylation and thermogenesis-related gene expression in both sWAT and eWAT. This type of movement seems to be the most effective exercise type for treating adipose tissue in diabetes [65].

In 2023, Azizi et al. [66] investigated the effect of ST on inflammatory responses and apoptosis in the pulmonary tissue of mice with T1D. The researchers divided the mice into four groups: control, control + Swimming, diabetic, and diabetic + swimming, with eight mice per group. Two weeks after the STZ injection to induce diabetes, a four-week swimming exercise intervention was implemented. The study found that T1D significantly elevated IL-1β, Bax, and Cas-3, while decreasing the anti-apoptotic marker Bcl-2. Swimming exercise demonstrated a protective impact, reducing the thickness of the inter-alveolar septum and the mean alveolar area [66].

**Figure 3:**
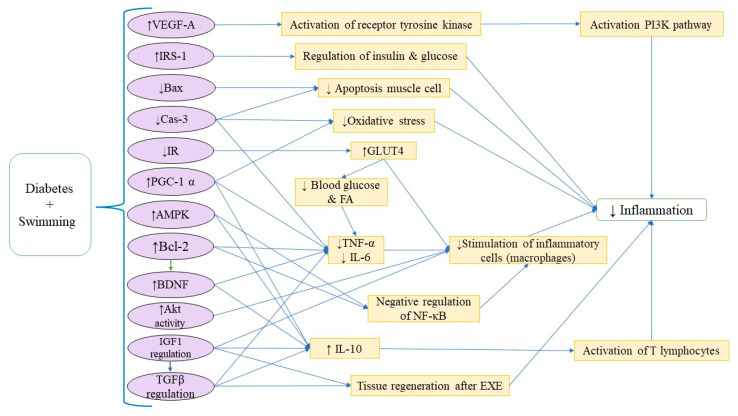
The effect of regular swimming practice on inflammation in diabetic patients. Regular swimming training in diabetic patients regulates TGF-β and IGF-1; reduces IR, Cas-3, and Bax; and increases PGC-1α, AMPK, Bcl-2, BDNF, IRS-1, and VEGF-A. Then, by negatively regulating NF-κB, reducing TNF-α and IL-6, increasing IL-10, and activating T lymphocytes, inflammation is reduced. Reducing insulin resistance increases GLUT4, lowers blood glucose and fatty acid levels, and reduces TNF-α and IL-6, thereby helping to reduce inflammation by decreasing the stimulation of inflammatory cells. Also, increasing PGC-1α helps reduce inflammation by reducing oxidative stress. Increasing IRS-1 reduces inflammation by regulating glucose and insulin. Swimming increases VEGF-A by activating tyrosine kinase receptors and activating the PI3K pathway, which generally reduces inflammation. Abbreviations: Protein kinase B (Akt); AMP-activated protein kinase (AMPK); Bcl-2-associated X protein (Bax); B-cell lymphoma-2 (Bcl-2); brain-derived neurotrophic factor (BDNF); Caspase-3 (Cas-3); Glucose transporter type 4 (GLUT4); insulin-like growth factor-1 (IGF-1); interleukin-6 (IL-6); interleukin-10 (IL-10); Insulin receptor substrate 1 (IRS-1); nuclear factor kappa light-chain-enhancer of activated B lymphocytes (NF-κB); peroxisome proliferator-activated receptor gamma coactivator-1 alpha (PGC-1α); Phosphoinositide 3-kinase (PI3K); transforming growth factor-beta (TGF-β); tumor necrosis factor-alpha (TNF-α); Vascular endothelial growth factor A (VEGF-A); ↑: increase; ↓: decrease.

Ya et al. (2022) [67] conducted a study to examine the regulatory role of the angiotensin II/TGF-β1/Smad2 signaling pathway in diabetic myocardial fibrosis. The study was designed using swimming as an intervention in 40 male Sprague Dawley rats, divided into a diabetes model group (*n* = 30) and a control group (*n* = 10). Diabetes was induced by the diet and STZ. Exercise intervention was administered to 15 randomly selected rats in the diabetes group, consisting of non-weight-bearing swimming for 60 min/day, six days/week, for 8 weeks. Results showed elevated FBG levels and IR index in the diabetic rats, but the indices were lowered in the exercise group. Notably, the exercise group had significantly lower myocardial fibrosis. Moreover, the expression of type I collagen and TGF-β1 was heightened in the myocardial tissue of the diabetic rats. In contrast, the diabetic exercise group exhibited decreased mRNA expression of both type I and III collagen fibers, as well as TGF-β1. These findings led the researchers to conclude that swimming exercise may effectively mitigate the development and progression of myocardial fibrosis in diabetes, potentially through the suppression of angiotensin II/TGF-β1/Smad2 pathways (Figure 3) [67].

A 2022 study by Nazari et al. [49] assessed the impact of swimming on hepatic factors influencing inflammation and MS using C57BL/6 mice with induced EAE. They administered a 6-week swimming regimen and found that EAE significantly elevated fetuin-A levels (3.5-fold) while decreasing AMPK and NAD + levels in the liver. The swimming intervention effectively lowered fetuin-A levels to those of the control group and substantially increased AMPK and NAD+ levels in EAE-induced mice. These results led the researchers to conclude that regular exercise may mitigate inflammatory responses and reduce MS severity, potentially through modulation of fetuin-A expression and enhancement of AMPK and NAD^+^ levels in hepatic tissue. This research highlights the potential therapeutic function of swimming activity in regulating MS-related inflammation and liver function [49].

In 2022, Habibi et al. [59] examined the positive impact of swimming activity on pancreatic histological damage in an OVX rat model of diabetes. This study aimed to investigate the effect of exercise on pancreatic function in a condition mimicking postmenopausal diabetes. The study’s findings revealed that estrogen insufficiency leads to increased inflammation of β-cells, which subsequently impairs insulin release. Notably, the researchers observed that swimming exercise effectively reduced inflammation in the postmenopausal diabetic model. According to the outcome, the team stated that swimming exercises may help alleviate inflammation associated with postmenopausal diabetes and aid in preserving pancreatic function after menopause. This investigation highlights the therapeutic aspect of swimming activity in managing pancreatic complications related to postmenopausal diabetes, suggesting it is a promising intervention for maintaining pancreatic health in this population [59].

A 2022 investigation by Eldesoqui et al. [68] examined the combined impact of dapagliflozin (a sodium-glucose transport-2 inhibitor) and swimming activity on diabetic cardiomyopathy (DCM) in Sprague Dawley rats. The researchers induced T2D using HFD and STZ, then divided the rats into the following groups: control, diabetic, diabetic with swimming, diabetic with dapagliflozin, and diabetic with both interventions. The combination of dapagliflozin and exercise exhibited the most pronounced cardioprotective effects, as evidenced by improved myocardial histopathology and reduced serum glucose, Creatine kinase MB (CK-MB), and LDH levels. This combined approach also decreased myocardial malondialdehyde, matrix metalloproteinase-9 (MMP-9), TNF-α, TGF-β, IL-1β, and the expression of Cas-3. Furthermore, it increased GSH, catalase, and insulin levels and upregulated microtubule-associated protein 1A/1B-light chain 3, indicating enhanced cardiac autophagy. The study concluded that the combination of dapagliflozin and exercise provided superior antioxidant, anti-inflammatory, and antifibrotic effects in DCM, while also enhancing cardiac autophagy, suggesting a potentially powerful therapeutic strategy for managing diabetic heart complications [68].

In 2022, Daghigh and Karimi [69] evaluated the impact of ST on fibrosis and inflammatory markers in the lungs of female OVX diabetic rats. The researchers used 40 rats, divided into four groups: sham (surgery without ovariectomy), OVX (ovariectomy), OVX + diabetic (OVX rats with HFD-induced diabetes), and OVX + diabetic + exercise (OVX diabetic rats subjected to eight weeks of ST). The study’s outcome demonstrated significant variety in protein expression between the exercise group and the OVX diabetic group without exercise. These results demonstrated the strong potential of swimming training in ameliorating lung conditions in estrogen-deficient diabetic subjects. The research highlights the possible therapeutic benefits of swimming exercise in managing lung-related complications associated with postmenopausal diabetes, suggesting that regular swimming could be an impactful treatment for maintaining lung health in this specific population [69].

da Silva Pereira et al. (2022) [70] investigated VEGF-A, IL-1β, TNF-α, and type I collagen expression in the placental tissue of diabetic rats performing swimming exercises. The study used 30 rats divided into six groups: non-diabetic controls, diabetic controls, and groups combining diabetes, exercise, and insulin treatment. STZ was used to induce diabetes. After 20 days, diabetic groups showed increased expression of inflammatory markers, VEGF-A, and type I collagen, along with higher placental apoptosis. The exercise-only diabetic group had reduced glycemia but did not protect placental tissue. Insulin-treated groups maintained similar levels to those of the controls. The researchers concluded that swimming exercise alone, despite lowering blood glucose, could not prevent placental changes [70].

Santos et al. (2021) [102] investigated sebaceous gland (SG) density in mice with diabetes and normal weight and the effects of N-acetylcysteine (NAC) and swimming on SG healing. The study used 25 mice divided into five groups: control, untreated diabetic, diabetic with swimming, diabetic with NAC, and diabetic with both swimming and NAC. Diabetic mice in the swimming group exercised for 30 min, five times a week, over a three-week period. The researchers found remarkable similarities between the control and diabetic swimming groups, whereas the untreated diabetic and NAC-treated groups showed potential differences. Adipophilin immunohistochemistry revealed intense lipid staining in SGs, whereas no changes were observed in the control group. Diabetic mice treated with NAC showed deformation. The combined swimming and NAC group showed mild SG recovery, while the swimming-only group demonstrated a more pronounced recovery. The researchers concluded that physical exercise enhanced tissue recovery despite diabetes-related challenges, suggesting potential therapeutic implications for managing SG function in diabetic conditions [102].

Sadeghian et al. (2021) [71] investigated the effects of genistein and swimming (alone and in combination) on retinal angiogenesis, OS, and inflammation in OVX Wistar rat models of diabetes, divided into the following groups: sham, OVX, OVX + diabetes, OVX + diabetes + genistein (1 mg/kg daily for eight weeks), OVX + diabetes + exercise (eight weeks), and OVX + diabetes + genistein in addition to exercise (eight weeks). After eight weeks, the OVX.D group showed elevated levels of matrix metalloproteinase-2, miR-132, miR-146b, NF-κB, extracellular signal-regulated kinases, TNF-α, VEGF, IL-1β, and MDA factor, with reduced GSH levels compared to rats in the sham and OVX groups. Genistein and exercise mitigated these diabetes-induced changes, with the combination showing greater effectiveness than either alone. The researchers suggested that the combined effect of exercise and genistein on microRNAs and their target proteins involved in inflammation, OS, and extracellular matrix metalloproteinase pathways may reduce the neovascularization of the retina [71].

Rahman et al. (2021) [72] reported that swimming exercise, alone or combined with melatonin, improved metabolic and neurobehavioral outcomes in a rat model of type 2 diabetes. Swimming enhanced glycemic control, reduced hippocampal inflammation, and increased BDNF and mitochondrial biogenesis markers, suggesting its potential to alleviate depression-like symptoms through improved metabolic and neuroinflammatory regulation [72].

Netto et al. (2021) [73] examined the effects of daily 30 min swimming sessions on salivary tissue inflammation in non-obese diabetic (NOD) mice. Thirty female mice were divided into five groups: controls, untreated NOD mice, and NOD mice receiving exercise alone or in combination with pharmacological therapy. Swimming exercise alone reduced lymphocyte infiltration, decreased inflammatory markers, and increased neutrophil counts, while also enhancing nuclear and cytoplasmic volumes and insulin receptor expression in salivary tissues. Importantly, physical exercise potentiated the therapeutic effects of drug treatments, indicating that regular swimming can modulate immune responses and reduce tissue inflammation [73].

Gilak-Dalasm et al. (2021) [74] assessed the impact of swimming activity on depression-like behavior in a mouse model of T2DM. The experiment utilized male C57BL/6 mice, and the condition was induced using HFD and STZ. Following the intervention, a reduction in depression-like behaviors was observed among the diabetic mice (e.g., anhedonia). Furthermore, the exercise regimen improved glycemic conditions and lowered inflammatory cytokines in the mice. Based on these observations, the researchers posited that swimming activity attenuated depression-like behavior in type 2 diabetic mice, potentially by reducing inflammation. This study proposed that frequent swimming activity may represent a potential non-pharmacological method for managing the metabolic and psychological complications of T2D [74].

Chen et al. (2020) [75] evaluated the role of long-term swimming activity on insulin sensitivity using a mouse model. The experimental protocol involved 60 min of swimming daily, 5 days/week, for eight weeks. The findings indicated that the long-term intervention significantly enhanced insulin sensitivity. This improvement was demonstrated through several measures, including HOMA-IR and glucose and insulin tolerance tests. At the same time, the intervention increased serum IL-4 and promoted the phosphorylation of insulin receptor substrate-1 (IRS-1) and protein kinase B (Akt). The researchers conducted in vitro experiments using skeletal muscle C2C12 cells to elucidate the underlying mechanism. The treatment also improved Akt phosphorylation and insulin-stimulated glucose uptake. Moreover, the administration of ruxolitinib, which inhibits Janus kinase, countered the increased insulin sensitivity triggered by IL-4 [75].

Alipour et al. (2020) [60] studied the effects of swimming on NF-κB and miR-146a in T2D in the pancreas of male rats. The study involved 28 male Wistar rats divided into four groups: control, exercise, diabetes, and diabetic exercise. The condition was induced using STZ and HFD. After induction of diabetes, the exercise groups followed a 12-week swimming regimen. Results showed that diabetic rats had higher levels of NF-κB, miR-146a, IL-6, IL-1β, and TNF-α, while pancreatic IRAK1 and TRAF6 were lower, and the treatment mitigated these differences. The findings suggest that swimming may benefit pancreatic inflammation in T2D by modulating key inflammatory mediators and signaling molecules [60].

Montenegro et al. (2019) [76] investigated the effects of swimming exercise on experimentally induced endometriosis in female Wistar rats. Seventy animals were divided into seven groups, performing light (once per week), moderate (three times per week), or intense (five times per week) swimming either before or after induction of endometriosis. The results showed that swimming reduced the size of endometriotic lesions, with the most significant effects observed in the moderate and intense exercise groups. Biochemical analyses revealed increased FAS levels and decreased MMP-9 and PCNA expression, alongside a reduction in oxidative stress. These findings suggest that regular swimming can modulate inflammatory and oxidative processes, highlighting its potential as a systemic anti-inflammatory intervention [76].

Korb et al. (2018) [99] conducted an investigation into the impact of regular aerobic exercise on cytokine levels and histone deacetylase (HDAC) activity in the peripheral blood of human subjects with T2DM. Twenty-one patients (10 males and 11 females) were recruited and subjected to a 12-week periodized training program involving walking/running on land or in water. The findings revealed that acute exercise-induced an uptick in HDAC activity in sedentary individuals after the 12-week training period. However, HDAC activity decreased subsequent to treatment. Furthermore, the 12-week periodized exercise program in both environments resulted in elevated IL-10. Overall, the findings suggest that the beneficial effects of exercise on diabetes symptoms may arise through altered HDAC activity and reduced inflammation. Notably, the study demonstrated that exercise led to a similar positive impact on epigenetics and inflammation in the subjects. This research has demonstrated the molecular mechanisms underlying the activity benefits in T2DM management, suggesting that swimming and land-based activity programs may effectively modulate key biological markers associated with the disease. The limitations of human studies in this field hinder the connection between animal research and clinical outcomes. However, future studies in this subject could enhance clinical applications [99].

Rahman et al. (2017) [77] studied the combined impact of physical activity and melatonin on hypertension, IR, and fatigue syndrome in T2DM rat models. The experimental design comprised five groups: normal control (NC), T2DM control (DC), diabetes plus exercise (DE), diabetes plus oral melatonin supplement (DM), and diabetes plus melatonin and exercise (DME). The intervention protocol involved oral melatonin administration (5 mg/kg, twice daily) and swimming exercise (40 min/day, 5 days/week). The findings revealed that the DC group exhibited significant increases in insulin, IR, FBG, BP, lipid profiles, serum leptin, lipid peroxidation, and inflammatory cytokines. Concomitantly, a decline was observed in antioxidant activity, exercise performance, and serum adiponectin levels. The combined melatonin and exercise intervention (DME group) markedly attenuated hypertension, IR, and diabetes-induced biochemical alterations while significantly enhancing exercise performance. Furthermore, the DME group demonstrated upregulation of GLUT4 and PGC-1α, as well as nuclear respiratory factors and mitochondrial transcription factor A, in muscle tissue. Based on these observations, the researchers proposed that the combination of melatonin supplementation and physical activity may improve hypertension, IR, and fatigue in T2DM rats, potentially through the improvement of physiological response to oxidative stress, lowering hyperlipidemia, and suppressing inflammatory cytokines [77].

Motta et al. (2016) [78] evaluated HIIT in C57BL/6 mice with high-fat diet–induced obesity. Exercise reduced body weight, improved glucose tolerance and lipid profiles, decreased hepatic steatosis, and lowered plasma inflammatory cytokines. HIIT also enhanced hepatic beta-oxidation, increased PPAR-α and GLUT4, and decreased PPAR-γ levels, demonstrating that regular exercise mitigates metabolic dysfunction and inflammation associated with obesity [78].

Ghiasi et al. (2016) [62] studied the impact of frequent swimming exercise on serum levels of CRP, IL-6, and TNF-α in rats with HFD-induced diabetes. Male Wistar rats were randomly divided into four groups: control (*n* = 10), diabetic (*n* = 10), exercise (*n* = 10), and diabetic exercise (*n* = 10). Through HFD and 35 mg/kg of intraperitoneal STZ, diabetes was induced. Following the induction of diabetes, the exercise groups participated in a 10-week swimming regimen (60 min, 5 days/week). Following treatment, monocytes and lymphocytes proliferated, while neutrophils declined in number. These elements were remarkably reversed to control levels through the swimming intervention. Elevated CRP, TNF-α, and IL-6 were observed in the diabetic group and were ameliorated by swimming. Histopathological analysis suggested that swimming may mitigate diabetes-induced damage. The researchers concluded that this intervention is related to improvements in inflammatory responses and mediators and pancreatic health in this model [62].

Kesherwani et al. (2015) [79] investigated the effects of daily one-hour swimming sessions, five days per week for eight weeks, on cardiac dysfunction in high-fat diet–induced obese C57BL/6J mice. Exercise attenuated weight gain, reduced TNF-α, increased IL-10, and decreased cardiac fibrosis. Functional cardiac parameters, including fractional shortening and ejection fraction, were improved in exercised mice, highlighting swimming’s ability to mitigate obesity-related inflammation and cardiac impairment [79].

In a 2011 study, Teixeira De Lemos et al. [80] assessed short-term exhaustive exercise and long-term low-intensity training in obese ZDF rats over a 12-week period. Acute exhaustive exercise reduced glycemia and insulinemia but increased inflammatory and oxidative stress markers, whereas long-term training improved glycemic control, lipid profiles, and lowered inflammation and oxidative stress. These results highlight that sustained, moderate exercise can mitigate metabolic dysfunction and systemic inflammation in obesity and diabetes [80].

A 2009 study by Teixeira de Lemos et al. [63] investigated the impact of aerobic exercise on inflammation in obese and lean ZDF rats. After 12 weeks of swimming, diabetic ZDF rats showed reduced hyperuricemia, TNF-α, and IL-6 levels. Exercise also normalized pancreatic weight and reduced pancreatic IL-6 and TNF-α expression, supporting the anti-inflammatory benefits of aerobic exercise in diabetes [63].

Teixeira De Lemos et al. (2007) [81] examined the impact of ST on inflammatory markers and IR in ZDF rats. The study utilized 8-week-old male ZDF Gmi *fa*/*fa* and Gmi^+/+^ rats, along with their littermates. Subjects were randomly allocated to either an exercise group or a sedentary control group. The exercise protocol consisted of swimming for 60 min/day, 3 days/week, over 12 weeks. In the exercised ZDF (*fa*/*fa*) rats, researchers observed complete or partial prevention of metabolic abnormalities, including hyperglycemia, hyperinsulinemia, and dyslipidemia, compared to their sedentary counterparts. Notably, despite no significant changes in body weight, the exercise intervention resulted in a 28.0% increase in plasma adiponectin and a 12.7% decline in CRP levels. It was demonstrated that the 12-week ST program was associated with improvements in long-term inflammation markers, as indicated by changes in adiponectin and CRP levels. Furthermore, the study suggested that the insulin sensitivity triggered by an exercise regimen may be related to these changes in inflammatory mediators (Figure 3) [81].

### 3.9. Cardiovascular Disorders

Sharma et al. (2018) [82] investigated the cardioprotective effects of copper nanoparticles (CuNP) and exercise, alone and in combination, in the treatment of myocardial infarction (MI) induced by isoproterenol (ISO) in an animal model. The study protocol involved the oral administration of 1 mg/kg/day of the nanoparticles for 4 weeks, along with swimming activity (90 min, 5 days/week for 4 weeks). The researchers assessed cardio-protection by measuring serum nitrite/nitrate concentrations and reductions in cardiac injury markers (cTnI, CK-MB, LDH), as well as alterations in the lipid profile, oxidative stress, and anatomical deformities. Results showed that ISO treatment significantly increased cardiac injury markers, altered lipid profiles, increased oxidative stress, and decreased serum nitrite/nitrate concentrations. Both CuNP and exercise treatments, both separately and in combination, significantly reduced ISO-induced cardiac injury markers and improved nitrite/nitrate concentrations and lipid profiles. The treatments also demonstrated a preconditioning effect against oxidative stress, offering protection against structural abnormalities. Importantly, the cardioprotective effects were associated with increased phosphorylation of glycogen synthase kinase-3 beta (GSK-3β) and Akt. The authors concluded that at low doses, copper nanoparticles and exercise remarkably prevent ISO-induced MI via GSK-3β inhibition and preconditioning, with the combined strategy showing enhanced potency due to increasing NO levels, improving lipid profiles, and reducing oxidative stress [82].

de Souza et al. (2021) [45] found that swimming training normalized BP in high-sodium (HS)-fed Wistar rats. After 22 weeks on a 2% NaCl diet, swimming reduced sympathetically related pressor activity and protected renal function, thereby mitigating glomerular contraction. Swimming decreased elevated CSF sodium levels in HS rats, with no blood volume differences. The authors concluded that swimming normalizes BP in HS rats via neurological processes modulated by CSF Na+ and renal protection, suggesting it can mitigate cardiovascular and renal harm from high sodium intake [45].

Tso et al. (2020) [103] examined the relationship between NSAID use and risk factors for cardiovascular disease in American-style football players. A number of football players, 60 endurance athletes, and 63 non-athletic controls were recruited and subjected to echocardiography, vascular application tonometry, and clinical assessments before and after one athletic season. Results showed that ASF athletes experienced elevation in weight, systolic blood pressure (SBP), and pulse wave velocity, along with decreased diastolic function (E’). NSAID use among football players increased in step with post-season SBP and weight gains, with daily users exhibiting the maximum post-season SBP and weight, in contrast to “never/rare” users. After regulating various factors, an association appeared between more frequent NSAID use and increased weight. The researchers concluded that habitual NSAID use, which often starts in adolescence, is linked to cardiovascular risk (particularly increased weight) in football players. This study underscores the potential long-term health implications of NSAID use in young athletes and suggests a need for more accurate monitoring and education regarding NSAID consumption in this population [103].

Chen et al. (2018) [83] investigated the preservative effects of physical activity on the cardiac conditions associated with age using a D-galactose-induced aging model in Sprague Dawley rats. The study employed a swimming exercise protocol consisting of 60 min sessions in warm water five days per week. Results demonstrated that the aging rat hearts exhibited cardiomyocyte disarrangements, which were notably improved by long-term exercise training. The exercise regimen upregulated key proteins that play a crucial role in cellular energy metabolism and longevity, including SIRT1, PGC-1α, and the AMP-activated protein kinase alpha-1 subunit. Additionally, exercise training suppressed the expression of aging-associated inflammatory cytokines. Based on the outcome, the researchers concluded that chronic physical activity may potentially improve SIRT1-associated anti-aging signaling pathways, thereby providing cardioprotection against aging-related cardiac changes. This work underlines the critical role of repeated physical activity as a potential intervention to reduce age-related cardiac deterioration and supports the role of SIRT1-mediated pathways in exercise-induced cardioprotection.

Santos et al. (2016) [104] investigated the impact of pre-existing exercise training on post-myocardial infarction (MI) outcomes in rats, focusing on the importance of PPAR-α in modulating the inflammatory response. The study utilized a 1-h/day swimming regimen, 5 days/week, for 8 weeks prior to induced MI. Rats were allocated into exercise and sedentary groups, with assessments at 7 days (EI1, SI1) and 28 days (EI4, SI4) post-MI. Key findings included lower MI-related mortality in the EI4 group compared to SI4 despite similar MI sizes. The EI4 group demonstrated better ventricular function, evidenced by a higher shortening fraction and lower apoptosis rates in infarcted areas. Immunohistochemistry revealed lower TNF-α levels in EI1 compared to SI1 in areas involved in infarction. Interestingly, in non-infarcted areas, EI4 demonstrated elevated TNF-α concentrations and a positive association between NF-κB and PPAR-α, contrasting with SI4. The researchers concluded that pre-MI exercise training led to improved chronic ventricular function post-MI, reduced inflammatory markers on a local scale, and lowered apoptosis in myocardial tissue, potentially mediated by PPAR-α. This study underscores the cardioprotective benefits of regular exercise prior to cardiac events and suggests a role for PPAR-α in exercise-induced cardioprotection [104].

Kumral et al. (2016) [47] found that in RVH rats, exercise affected RVH-induced OS and cardiac issues. Sedentary RVH rats exhibited increased aortic contraction, enlarged left ventricles, elevated aortic iNOS, decreased aortic eNOS, reduced ejection fraction, higher levels of pro-inflammatory cytokines, increased lipid peroxidation, and lower cardiac antioxidant levels. However, RVH rats with a 9-week swimming regimen showed increased eNOS, decreased iNOS, and reversed echocardiographic/oxidative changes. They concluded that exercise reduces hypertension-related oxidative injury via endothelial NO system modulation, highlighting exercise’s therapeutic benefits for renovascular hypertension and cardiac complications [47].

Bulut et al. (2016) [84] examined how agonists of estrogen and oxytocin receptors can protect against myocardial injury in sedentary and exercised OVX rats. Sprague Dawley rats were divided into sham-operated (control) and OVX groups, with some rats undergoing a 4-week swimming exercise regimen. Treatments included saline, an estrogen receptor α agonist (propyl pyrazole triol [PPT]), an estrogen receptor β agonist (diarylpropionitrile [DPN]), or oxytocin. Results showed that resection of the ovaries led to anxiety and weight gain in inactive rats, while activity protected against weight gain. TNF-α levels, which were higher in OVX rats, were lowered by exercise or treatment with DPN, PPT, or oxytocin. IL-6 concentrations were reduced by all therapies when combined with physical activity. Cardiac muscle fiber disorder was diminished in all exercised rats. The researchers concluded that estrogen receptor agonists and oxytocin, particularly when combined with exercise, may offer practical new therapeutic approaches for protecting against myocardial ischemia in postmenopausal women. This study reveals the additive impact of hormone-based treatments and exercise in mitigating cardiovascular risks associated with estrogen deficiency [84].

Silva et al. (2015) [85] investigated the impact of propolis and swimming, both individually and in combination, on dyslipidemia, atherogenesis, and left ventricular hypertrophy (LVH) in LDLr^−/−^ mice with abnormally high cholesterol levels. Prior to random allocation into four groups, the mice were fed an HFD for 75 days and then allocated into four groups: a sedentary control group (HL), a swimming group (NAT), a propolis-treated group (PRO), and a combination group receiving both swimming and propolis (HL + NAT + PRO). The HL group demonstrated extreme dyslipidemia, atherogenesis, and LVH related to decreased serum HDL cholesterol levels and increased cardiovascular inflammation, as indicated by elevated CD40L expression in the left aorta and ventricle. Both treatments, independently or in combination, prevented atherogenesis, LVH, and inflammation in the ventricles and arteries. These interventions decreased the expression of CD40L and increased plasma HDL cholesterol levels. The researchers concluded that propolis, either alone or in combination with regular physical activity, offers cardiovascular preservation through anti-inflammatory action. This study highlights the potential of natural supplements, such as propolis, and exercise as complementary approaches to managing cardiovascular risk factors associated with hypercholesterolemia [85].

Preto et al. (2015) [86] used BALB/c with long-term Chagas disease as animal models to investigate the impact of mild physical activity on the right and left ventricles and evaluated the results using morphometric and stereological methods. Four-month-old mice (*n* = 20) were equally assigned to four groups: untrained control, trained control, untrained infected, and trained infected. The exercise protocol consisted of swimming for 30 min/day, five days/week, for eight consecutive weeks. The researchers found that this low-intensity aerobic exercise regimen led to remarkable alterations in the cardiac tissue of Chagasic animals in the trained infected group. Specifically, the exercise promoted an increase in capillary density and a decrease in collagen fiber density. Additionally, there was a reduction in the cross-sectional area of cardiomyocytes in the trained infected group. These indicate the positive influence of mild aerobic exercise on ventricular morphology and morphometry in *Trypanosoma cruzi*-infected mice, mitigating the pathological changes caused by the infection. Notably, the exercise diminished the cardiac parameter differences between infected mice and uninfected controls, indicating a potential therapeutic point of exercise in chronic Chagas disease [86].

Nounou et al. (2012) [87] evaluated the potential protective impacts of flaxseed supplementation and physical activity on lipid profile, cardiac health, and inflammation in a rat model of isoproterenol (ISO)-induced myocardial ischemia. The study utilized 40 male albino rats, which were allocated into five groups: a control group, an ISO-induced myocardial ischemia group, two groups with ISO-induced myocardial ischemia that received oral flaxseed supplements for six weeks as pre-treatment, and a group subjected to swimming exercise. The researchers observed alterations in lipid profiles, cardiac markers, and TNF-α, IL-1β, and pentraxin 3 (PTX 3) in the group suffering from myocardial ischemia. The combination of supplements and physical activity resulted in a significant increase in HDL and paraoxonase-1 levels, while troponin, TNF-α, and IL-1β levels were substantially reduced. Receiver operating characteristics analysis of PTX 3, cTnI, IL-1β, and TNF-α demonstrated adequate levels of specificity and sensitivity. The authors concluded that frequent exercise enhances the plasma concentration of lipoprotein and cardiovascular preservation provided by supplementation with flaxseed, which reduces atherosclerosis [87].

Shapoval et al. (2011) [105] focused on medullary neurons to examine the hemodynamic impacts associated with regulating mitochondrial permeability transition and initiating neuronal nitric oxide synthase (NOS1) following moderate exercise. The study, conducted on urethane-anesthetized rats that underwent a four-week daily swimming regimen, suggests that physical training may have a preservative effect on the practical activity of medullary neurons, potentially due to lowered sensitivity of the mitochondrial permeability transition pore (MPTP) to opening. In trained rats, injections of melatonin, an MPTP opening inhibitor, into specific medullary neuronal populations resulted in a reduction in systemic arterial pressure, contrasting with the primarily hypertensive responses observed in untrained animals. Additionally, injections of L-arginine, a neuronal nitric oxide (NO) synthase (NOS-1) activator, into the medullary nuclei of exercise-trained rats elicited more pronounced hemodynamic shifts compared to control animals, suggesting a rise in neuronal NO synthase activity within the medullary neurons of trained animals. It was proven that moderate physical training may influence the functional characteristics of medullary neurons, potentially through mechanisms involving mitochondrial permeability and nitric oxide synthesis [105].

Baptista et al. (2008) [88] investigated the impacts of two distinct chronic conditions with medium-intensity aerobic training protocols, swimming and running on a treadmill, on cardiovascular physiological adaptations in normal Wistar rats. Both exercise groups demonstrated increased HDL cholesterol levels compared to the sedentary control group. The study observed enhanced ADP-induced platelet aggregations in rats in the exercise group, accompanied by elevated mean platelet volume and platelet distribution width. Red blood cell patterns were altered in both training groups, with the swimming group exhibiting remarkably elevated cell counts and hematocrit, and reduced mean corpuscular hemoglobin and mean corpuscular hemoglobin concentration values, indicating red blood cell restoration. Plasma and platelet serotonergic system levels were elevated in both exercise groups. Notably, the levels of norepinephrine and epinephrine in plasma showed a markedly higher increase in the swimming group. The researchers concluded that while both aerobic exercise protocols yielded similar beneficial effects on lipid profiles, they produced distinct cardiovascular physiological adaptations. Treadmill running appeared to have a greater influence on peripheral serotonergic system modulation, whereas swimming demonstrated a more pronounced effect on sympathetic nervous system (SNS) activation. This evidence suggests the need to consider the specific type of aerobic exercise when evaluating its impact on cardiovascular adaptations [88].

Nunes et al. (2008) [89] studied the effects of exercise on hemodynamics, muscle lipid peroxidation, and plasma IL-10 levels in 40 rats with chronic heart failure (CHF). The subjects were allocated to four groups: trained CHF (T-CHF), sedentary CHF (S-CHF), trained sham (T-Sham), and sedentary sham (S-Sham). Trained groups underwent a swimming protocol for 60 min/day, 5 days/week, for 8 weeks. The 8-week exercise program improved diastolic function in the T-CHF group, as indicated by a reduction in left ventricular end-diastolic pressure compared to the S-CHF group. Lipid peroxidation was higher in the S-CHF group, with no significant difference between the T-CHF, S-Sham, and T-Sham groups. Plasma IL-10 levels were lower in the S-CHF group compared to all other groups. The researchers concluded that regular exercise improved cardiac function, reduced inflammation, and decreased muscle damage in CHF rats, emphasizing the benefits of structured exercise for managing chronic heart failure [89].

Alkatan et al. (2016) [92] investigated the impact of cycling and swimming on vascular function in patients with OA. Overall, 48 middle-aged and older OA patients were enrolled and randomly assigned to either cycling or swimming groups for a 12-week period. The exercise protocol began with 20–30 min/day, 3 days/week, at 40–50% of heart rate reserve (HRR), gradually increasing to 40–45 min per day at 60–70% of HRR. Results demonstrated significant decreases in central arterial stiffness and carotid artery stiffness, as well as reduced IL-6 levels in both swimming and cycling groups. However, other inflammatory markers remained unchanged. Notably, vascular endothelial function was enhanced remarkably after swimming but not after cycling. The researchers concluded that frequent swimming activity can produce comparable or greater impacts on inflammation and vascular function in comparison to cycling activity in OA patients. These findings suggest that swimming may be a particularly beneficial exercise modality for improving cardiovascular health in individuals with osteoarthritis [92].

### 3.10. Negative Effects of Swimming

Winter swimming, defined as deliberate immersion of the whole body in cold water, represents a significant physiological stressor. Habitual practitioners often exhibit varying degrees of cold adaptation. This recreational activity has garnered scientific attention due to its potential beneficial and deleterious health implications. Winter swimming may enhance stress tolerance and promote a phenomenon termed “body hardening”. For individuals with good baseline health who engage in frequent, gradual, and adaptive physical activity, this type of exercise appears to be beneficial for cardiovascular and overall health. Nevertheless, the activity is not without inherent risks, particularly for novice practitioners. These risks include the potential for mortality due to cold-shock response in the first stages of adaptation, gradual loss of swimming efficiency, or the risk of hypothermia. Moreover, as with any intense physical exertion, individuals with overt or occult cardiovascular conditions may be at elevated risk for adverse effects, including arrhythmias and acute cardiovascular events. Consequently, a gradual, stepwise approach to initiating and developing this activity is suggested in order to facilitate acclimation, mitigate risks associated with exposure, and potentially derive health benefits. Further research, particularly prospective longitudinal studies, is imperative to elucidate the acute and chronic health implications of this significant recreational activity [106].

Llorens-Martín et al. (2016) [107] investigated the effects of acute stress, utilizing the Porsolt forced swimming test, on hippocampal neurogenesis and inflammation, two key pathophysiological aspects of major depression. The study focused on the hippocampus, a region significantly affected by mood disorders. Utilizing PSD95-GFP-expressing retroviruses, the researchers observed that forcing young neurons to swim altered their dendrites and hindered the formation of connections between neurons, as evidenced by reduced postsynaptic densities. The acute stress also induced morphological changes and increased CD68 expression in microglial cells, suggesting their activation. Additionally, the study revealed notable alterations in the pattern of inflammation in the hippocampus, with changes in molecules such as IL-6 and eotaxin, which have been previously connected with mood disorders like depression. These results shaped our understanding of the various mechanisms underlying the hippocampal response to acute stress and their potential relevance to mood disorders, highlighting the complex interplay between stress, neuroplasticity, and inflammation in the context of depression [107].

Shi et al. (2012) [43] explored the impact of swimming in cold water on the development of a gouty arthritis model that mimics clinical pathogenesis. The researchers induced a persistent hyperuricemic state in rats by administering oxonic acid and feeding them hypoxanthine. During model preparation, the experimental group underwent daily cold-water swimming sessions at 10–12 °C for 10 min over a 12-day period. Results indicated that uric acid concentrations in the joint cavity were elevated in all groups receiving treatment, compared to the controls, with the hyperuricemia + cold water swimming group showing significantly higher levels than the other groups. Notably, only rats in the hyperuricemia + cold water swimming group exhibited ankle injuries characteristic of gouty arthritis, including hemangiectasis, congestion, and inflammatory cell infiltration in the synovium and surrounding soft tissue. These pathological changes were absent in other groups. The study concluded that cold water swimming can induce swelling similar to gouty arthritis with clinical characteristics in rats with persistent hyperuricemia, proposing a probable role for environmental factors in the precipitation of gout symptoms in predisposed individuals [43].

Gatmaitan et al. (1970) [108] assessed the effects of exercise on the virulence of murine coxsackievirus B-3 with respect to myocardiopathy. The study induced the condition in recently weaned mice through intraperitoneal and intracerebral inoculations of the Nancy strain, resulting in a short-term mortality rate of 5.5%. The cardiomyopathy manifested in two distinct stages: an initial stage of approximately 9 days characterized by necrosis in the myocardium, inflammatory responses, and the calcification and fibrosis associated with healing, and a later stage exhibiting continuing myocardial inflammatory lesions but no detectable infectious virus. When infected mice were forced to swim during either phase, virulence was significantly reduced. Fifty percent of the exercised mice succumbed to congestive failure, primarily during swimming, with cardiac examinations revealing dilation, hypertrophy, and gross necrosis. The myocardium exhibited complete necrotic transformation, with inflammatory and calcifying manifestations. Notably, the replication rate of coxsackievirus in the myocardium increased 530-fold in nurslings that were forced to swim during the peak infectious phase. The exercised mice also demonstrated an increased frequency of myositis in hind limbs and more severe inflammatory lesions in the adipose tissue surrounding the heart and kidneys. When swimming was initiated on the 9th day after infection, a moderate rise in virulence and lethality was observed. These findings elucidate the significant impact of exercise stress on the progression and severity of viral myocardiopathy in this murine model [108].

## 4. Current and Future Perspectives

Physical inactivity has been identified by the World Health Organization as the fourth leading cause of mortality globally, accounting for 5.5% of deaths worldwide. Conversely, physical activity has been linked to numerous health benefits, including an enhanced quality of life and a reduced risk of various pathologies, such as cerebrovascular accidents, hypertension, myocardial infarction, obesity, and certain types of neoplasms. The positive impacts of exercise on bone metabolism are attributed to intermittent exposure to myokines, including irisin, IL-6, leukemia inhibitory factor, and insulin-like growth factor-1. When released in an intermittent pattern during physical exertion, these myokines promote osteogenic activity. However, it is noteworthy that chronic elevation of these same elements can function as inflammatory or pro-resorptive mediators. Additionally, exercise-induced reductions in circulating levels of adipokines, such as leptin, visfatin, adiponectin, and resistin, further support the advantageous impacts on bone metabolism and contribute to an improved overall metabolic profile. This dual mechanism of exercise, through both the modulation of myokines and the reduction of adipokines, elucidates the complex interplay between exercise and metabolic homeostasis [109].

Regular physical activity may contribute to attenuating potential neurological impairment and chronic pain through neuroimmune modulatory mechanisms. This preventive effect is hypothesized to operate by influencing neural plasticity and inflammatory processes, thereby potentially reducing the severity and duration of neuropathic conditions [110]. Research has demonstrated the significance of exercise-induced analgesia in mitigating pain associated with various conditions, including fibromyalgia, low back pain, neuropathy, and osteoarthritis. Studies indicate that multiple endogenous systems are activated during and after physical activity, releasing factors and neurotransmitters such as opioids, nitric oxide, serotonin, catecholamines, and endocannabinoids. These biochemical agents are considered to have a major role in modulating pain perception. The activation of these systems and the subsequent release of pain-modulating substances suggest a potential mechanism by which exercise may contribute to pain relief. However, the precise mechanisms underlying this phenomenon and its full therapeutic potential require further investigation to elucidate the extent of its analgesic effects across different pain conditions [111]. Emerging evidence suggests that cold-water swimming (CWS) may play a potentially beneficial role in the amelioration of peripheral neuropathic pain and the promotion of functional recovery in the rat model [34], but it induces swelling that is similar to gouty arthritis [43]. The lack of human studies on neurological impairments complicates comparisons with animal studies and limits the inference of results.

Physical exercise has demonstrated potential neuroprotective properties, with evidence suggesting it may mitigate AD risk and enhance synaptic plasticity [23]. According to the studies reviewed in the previous section, we found that swimming, especially with moderate intensity [23]:

- Reduces inflammation, activates the TAN1/PI_3_K/CREB signaling pathway, decreases miR-34a levels, and preserves neuronal function and survival by preventing extra demyelination and inflammatory infiltration in the CNS [23].
- Significantly enhances axon regeneration and neuronal creation in motor neurons [21].
- Improves AD-induced alterations in α7nAChR, NLRP1, memory, and dark cells [24].
- Improves the neurogenesis and behavioral performance in adult neurogenesis mouse models of AD [25].
- Normalizes the hippocampal FNDC5/irisin expression (related to the reduced soluble β-amyloid peptide and phosphorylated tau protein, improved BDNF and insulin signaling proteins, and corresponding mitigation of cognitive impairments) [26].
- Increases BDNF, lowers both glutamate hippocampal concentration and TNF-α, and decreases neurobehavioral dysfunctions in patients with AD [27].

Research has shown that STZ injection in male rats, used as a model for AD, increases several pathological markers in the CA1 region of the hippocampus. These include reactive gliosis, proinflammatory cytokine release, oxidative damage, amyloid beta levels, tau hyperphosphorylation, Nrf2 protein expression, DNA binding activity, and the expression of downstream antioxidant genes. However, subsequent exercise interventions have been observed to attenuate these effects significantly. Exercise appeared to confer neuroprotection and suppress neuronal apoptotic-like cell death in this model [28].

The main problems PD patients face that prevent normal swimming are difficulty moving the limbs, poor coordination, and maintaining a horizontal position during swimming [112,113]. Also, PD increases the level of anxiety while swimming [112,114]. The therapeutic effects of antioxidants may increase swimming activity [112]. An investigation into the impact of swimming activity on Parkinson’s rats/mice models induced by 6-hydroxydopamine (6-OHDA) showed that swimming leads to a striking elevation of calretinin-positive interneurons in the striatum of Parkinsonian rats [32,115]. Calretinin has a major impact on the neuroprotective mechanisms of activity in PD [32]. Eventually, we observed that studies on PD disease were concordant, confirming that swimming is efficacious in improving neurological diseases, such as PD, due to its antioxidant and anti-inflammatory characteristics, as well as its ability to reduce cognitive and motor declines, depression, OS, and neuroinflammation caused by 6-OHDA [57]. However, it is essential to note that these benefits require confirmation through human studies, as findings from animal research cannot be directly applied to clinical practice.

CWS significantly increases motor and sensory deficits caused by removing the brachial plexus (BPA), reducing inflammatory cell infiltration, and vacuole creation in damaged nerves. Additionally, CWS enhances function and pain modulation in the early stages of BPA, accompanied by inflammatory suppression and spinal modulation [34]. However, there are limited human studies regarding the effects of swimming on neuropathy, and further investigation is necessary.

Swimming exercise can reduce the number of inflammatory cells in the blood of aged mice with knee osteoarthritis and improve the articular chondrocytes, matrix composition, and morphology of cartilage tissue. Thus, mice that swim will have smooth articular surfaces, normal chondrocytes, and less proteoglycan loss [37]. Additionally, swimming reduces pain and joint stiffness, as well as the development of histological and radiological symptoms of osteoarthritis in the knee joints. It also decreases serum CRP levels, tissue cartilage oligomeric matrix protein levels, as well as miR-130a and HDAC3 [38]. Given the elevated risk of cardiovascular disorders in patients with OA, frequent swimming activity, compared to land-based cycling exercise, can have similar or even superior impacts on vascular function and inflammatory markers [92]. Swimming reduces edema and does not elevate inflammatory infiltrates or cause congestion [39]. However, treadmill training is more impactful than swimming in reducing the activity of pro-inflammatory cytokines (IFN-γ, TNF-α, IL-1β, and IL-6), while positively regulating anti-inflammatory cytokines such as IL-4, IL-10, and TGF-β. Treadmill training also results in more satisfactory morphological outcomes regarding the number of chondrocytes in the histological formation [40].

Swimming benefits women with RA by improving disease activity, pain, and function [94]. Studies in female Wistar rats with type II CIA showed that swimming can modulate immune response and hormone levels, potentially counterbalancing arthritis’s effects [42]. These findings suggest that exercising in water in patients with RA reduces pain, improves performance, and balances the immune system’s cells. A study indicated that swimming in cold water can lead to swelling resembling gouty arthritis, with clinical features observed in rats with persistent hyperuricemia [43]. However, further studies are required to better understand the impact of swimming on gout.

Also, moderate swimming in aged rats reduced plasma creatinine, blood urea nitrogen, renal injuries (fibrosis, OS, triglycerides), and mRNA expression of actin alpha 2, Fn, collagen (type I and IV), and TGF-β1. It inhibited NF-κB, lowered pro-inflammatory cytokines (MCP-1, IL-1β, IL-6), and activated PPAR-α [44].

Swimming training effectively normalizes the BP of rats on a high-sodium diet, separately from its impacts on hydro-electrolytic stability, which may involve neurogenic mechanisms regulated by Na^+^ levels in the CSF, along with renal preservation [45]. Swimming also protects the liver, heart, and kidneys against doxorubicin toxicity by elevating antioxidants and lowering OS and inflammatory responses [46]. Regular swimming exercise after RVH surgery alleviated renovascular hypertension-induced oxidative injury by regulating oxidant–antioxidant balance through the activity of the endothelial nitric oxide (NO) system [47]. Regular moderate-intensity swimming for at least 6 weeks protects the kidneys from inflammatory and toxic damage and improves renal BP.

Sports training has been confirmed as having a remarkable and chronic effect on body weight [57]. NAFLD is one of the primary metabolic syndromes and initiators of chronic liver disorders, and can represent a wide range of liver pathologies [50]. In addition, evidence has shown that physical inactivity and Western dietary habits may facilitate the development of NAFLD [50]. On the contrary, physical activity suppresses tumor initiation and progression and positively impacts obesity and obesity-related co-morbidities via various mechanisms [57]. However, whether the nervous system mediates the suppressive effects of physical activity on tumors via increased DA levels remains unknown [53].

Investigations into the physiological effects of swimming training (ST) have revealed its influence on hepatic biomarkers. ST has been demonstrated to modulate levels of fetuin-A, AMPK, and nicotinamide adenine dinucleotide (NAD^+^) in liver tissue. Specifically, upregulation of AMPK and NAD^+^ concentrations has been observed concomitant with a reduction in fetuin-A levels [49]. Training followed by intense exercise boosts sIgA and pIgR concentrations in the proximal intestine. Also, distal intestinal segment sIgA increased, while sIgA and pro-inflammatory pIgR-producing cytokines mRNA downregulated [51].

In oncological contexts, both moderate-intensity swimming and dopamine agonist (DA) administration have demonstrated inhibitory effects on TGF-β1-induced EMT in transplanted hepatic carcinoma cells. Moderate exercise appears to suppress the progression of hepatic neoplasm via enhanced DR2 activity, whereas excessive exercise exhibits contrary effects [53]. Long-term swimming regimens have been associated with enhanced cognitive function and attenuated protein carbonyl and proteasome activity in the cerebral tissue of middle-aged and senescent rats [54]. Moreover, both acute and chronic swimming protocols have been correlated with significant reductions in body mass, visceral adiposity, and hepatic lipid accumulation in NAFLD models [50,55]. Swimming exercise has demonstrated efficacy in ameliorating IR and inflammatory cascades and may enhance lipid metabolism in IR and NAFLD pathologies, potentially via PPAR-γ-mediated signaling pathways in hepatic tissue [55].

Swimming training can reduce the destructive impacts of an HFD with a sedentary lifestyle (overweight, increased body mass, hyperglycemia, hyperinsulinemia with insulin resistance, adipocyte hypertrophy, pancreatic islet hypertrophy, dyslipidemia, changes in hepatic enzymes, and elevated inflammatory cytokines), and reduce NAFLD by changing gene expression of liver lipogenic and oxidative proteins in mice. These data reinforce the notion that swimming activity can be regarded as an effective non-pharmacological treatment for NAFLD, obesity, and insulin resistance [27]. In addition to the negative effects of an HFD that were investigated in previous studies, a fructose-enriched diet also has adverse effects and elevates oxidative damage, degeneration, inflammatory response, and necrosis in the liver, while swimming activity restores these impacts to a large extent [56] and significantly alleviates NAFLD [50].

Swimming, silymarin, and vitamin C significantly decrease hepatic inflammation markers (TNF-α, IL-1β), oxidative stress, liver inflammation, and fat accumulation and regulate hepatic enzymes while boosting antioxidant capacity and PPAR-γ [6]. Vitamin D and physical activity counter HFD-induced weight gain, steatosis, and inflammation and improve lipid profiles by reducing FATP-4, TLR-4, and inflammatory responses [57]. Finally, we concluded that regular moderate-intensity ST is beneficial for the liver and its related diseases, but high-intensity training has adverse effects. Conducting clinical trials could contribute to enhancing the application of this method in clinical practice.

The only study that identified the effects of swimming on the reproductive system reported that moderate-intensity swimming mitigates the adverse effects of being overweight on male reproductive function by reducing testicular OS and inflammation. In contrast, high-load exercise did not demonstrate such benefits [58].

Elevated inflammatory responses of β-cells impair insulin release in estrogen insufficiency, and swimming exercise eliminates inflammation in post-menopausal diabetes and has the potential to prevent pancreatic activity after menopause [59]. Also, swimming exercise protects the pancreas from the adverse effects of stress by different mechanisms through its anti-inflammatory and antioxidant effects [61], and causes a significant decrease in expression levels of miR-146a, NF-κB, and inflammatory cytokines, and a considerable increase in pancreatic expression levels of TRAF6 and IRAK1 [60].

In diabetic ZDF (*fa*/*fa*) rats, swimming activity lowered hyperuricemia and IL-6 and TNF-α levels, sustained the weight of the pancreas around the normal range, and lowered the expression of TNF-α and IL-6 in pancreatic islet cells [63]. Therefore, swimming helps improve pancreatic damage and maintain pancreatic health by reducing inflammatory factors [62]. In general, aerobic activity is anti-inflammatory in nature [63].

The only investigation we found measuring the impacts of swimming on the cardiorespiratory system found that a three-year swimming training program increased SCRI by fourfold [98]. Other studies focused on water temperature, disinfectants, and swimming environments. For example, increasing swimming training hours in winter caused a significant increase in nasal congestion/rhinorrhea and cough, whereas no such effects were observed in summer [95]. Intense, chronic swimming training in indoor chlorinated swimming pools is associated with airway disorders [60], although it does not alter FeNO, lung function, SP-D, 8-isoprostane, several cytokines, or VEGF. The increased airway permeability observed in healthy adults is due to exposure to disinfection byproducts without the involvement of inflammatory mechanisms [97].

T2DM often causes DCM, worsened by sedentary habits and HFDs [68,79]. DCM features myocardial OS, inflammation, apoptosis, suppressed autophagy, ECM remodeling, and fibrosis [68]. Pro-inflammatory cytokines (TNF-α) increase, while anti-inflammatory cytokines (IL-10) decrease, impacting cardiac health in obesity and diabetes [79]. Also, inflammation connects T2D and depression [74]. IR underpins obesity, diabetes, and CVD, while aerobic exercise boosts IL-4, improving IR and metabolic disorders [75]. Moderate swimming exercise has the potential to improve cardiac dysfunction [79], enhance the body’s natural antioxidant defense [73], and reduce depression-related symptoms [74].

Compared to resistance training, severe interval activity (running and swimming) improves cardiovascular health by enhancing autonomic regulation, VO_2max_, plasma lipid profile, and catecholamine levels in boys with type 1 diabetes [100]. Twelve weeks of walking or running in a swimming pool increased IL-10 levels in patients with T2D [99].

Swimming in female diabetic rats during pregnancy reduces glycemic levels but does not prevent immunohistochemical changes in the placenta [70]. In OVX diabetic rats, it reduces retinal neovascularization impairment [69] and alleviates inflammation in post-menopausal diabetes [59].

In male T2DM rat models, swimming increases plasma insulin, HDL, tissue B-cell lymphoma-2 [70], the expression of TRAF6 and IRAK1 [60], lymphocytes, and monocyte counts [62], and the autophagic capacity of the heart [68]. Swimming also causes a remarkable decrease in expression levels of miR-146a, NF-κB, and inflammatory cytokines [60], as well as in neutrophils, CRP, IL-6, and TNF-α [62], oxidative stress, and the size of endometriotic lesions [76]. Moreover, it reduces metabolic syndrome, neuroinflammation, and corticosterone levels [72] and overall inflammatory processes [62,73].

In general, these changes cause a reduction in depression-like behavior [72], pancreatic damage induced by diabetes [62], and a lowered risk of age-related disorders [54], such as cardiovascular disorders, T2D, cancer, and neurological diseases [54]. They also improve the progressive decline of renal function, along with histological variations like glomerulosclerosis and interstitial fibrosis [44], help prevent the occurrence and development of diabetic myocardial fibrosis [71], ameliorate IR, hypertension, and exercise performance or fatigue [77], and potentiate the therapeutic function of drugs on tissue restructuring in the salivary glands of a diabetic male rat model [73].

According to studies conducted so far, swimming exercise has been shown to improve cardiovascular health, suggesting that long-term physical activity training may potentially enhance SIRT1-related anti-aging signaling and provide cardioprotection against aging [83]. Swimming exercise alone or in combination with CuNP reduces CK-MB, cTnI, LDH, and oxidative stress and prevents MI. It also improves nitrite/nitrate concentration and lipid profile [82]. Reduced stages of local inflammatory markers and myocardial apoptosis are associated with the presence of PPAR-α in exercised animals [104]. Regular ST generally benefits cardiovascular protection through anti-inflammatory action [85] and reduces muscle cell damage [89]. Also, by reducing the pathophysiology of atherosclerosis, it improves plasma lipoprotein levels and increases cardiovascular protection [87], and has similar or even more positive impacts on vascular function and inflammatory markers in comparison to cycling activity [92]. Running on a treadmill is more impactful than swimming in regulating the peripheral serotonergic system, while swimming is more critical in activating the SNS [88]. Low-intensity aerobic swimming training affects the morphological and morphometric parameters of the left and right ventricles and reduces the alterations resulting from the organism [86]. ST normalizes BP levels in male Wistar rats fed high sodium (HS) [45]. Also, regular ST after RVH surgery reduces vascular BP by regulating the oxidant–antioxidant balance through the presence of the endothelial NO system, which causes oxidative damage [47]. Estrogen receptor agonists, in addition to oxytocin, combined with physical activity, may represent promising new therapies for protecting against myocardial ischemia in postmenopausal women [84].

According to previous studies, regular swimming exercise helps reduce pain and body weight, improves body composition, positively influences the response of anti-inflammatory cytokines, reduces levels of IL-10, IL-4, and IL-1β, and leptin, supports cartilage health, and enhances overall quality of life. Swimming is considered one of the primary non-pharmacological interventions for regulating inflammatory diseases. It enables individuals to engage in aerobic activity with reduced joint stress [91]. Future clinical studies could examine these effects in more detail.

Despite being recognized for its physiological benefits, swimming as a therapeutic tool is hindered by numerous logistical and infrastructural barriers, limiting its widespread adoption in the clinic [113]. Pool availability is not uniform; conditions are often worse in some rural areas or low-income contexts, due to inadequate maintenance of aquatic facilities. In larger metropolitan areas, access may be impeded not only by membership fees but also by limited pool hours specifically for rehabilitation use and a lack of temperature-controlled or accessible pools for patients with physical limitations. These limitations result in inequitable participation among patients and make swimming interventions less practical at a population level [114].

Additionally, there may be reasons not to prescribe swimming in specific patient populations, necessitating individual screening before prescription. Uncontrolled cardiovascular disease, severe chronic obstructive pulmonary disease, unstable asthma, active infection(s), poorly controlled epilepsy, and open skin lesions pose elevated safety risks in aquatic environments [115,116]. Additionally, exposure to a chlorinated pool may increase airway inflammation or induce bronchial hyperreactivity, particularly in individuals with atopy or underlying respiratory conditions [117,118]. Accordingly, proper pool ventilation, water quality control, and medical supervision must be provided to reduce risks and ensure patient safety during aquatic therapy.

## 5. Concluding Remarks

The current review article aimed to establish the effects of swimming intervention on inflammatory and anti-inflammatory mediators in various clinical conditions, including neurological, rheumatological, metabolic, and systemic inflammatory diseases. Upon analyzing these selected studies, it was observed that swimming exercise protocols resulted in a significant shift in the inflammatory profile, characterized by an increase in anti-inflammatory mediators (IL-10, TGF-β) and a reduction in pro-inflammatory cytokines (TNF-α, IL-6, and IL-1β). Such inflammatory response control has been repeatedly linked to superior clinical outcomes, including decreased oxidative stress, improved tissue repair, and enhanced metabolic performance. According to these findings, swimming is a beneficial intervention for treating illnesses caused or exacerbated by inflammation.

One of the major benefits of swimming, which was mentioned in this review, is its role as a low-impact sport. Swimming tends to injure bones and joints less than other sports due to the buoyancy effect of water, making the activity quite suitable for patients with joint disorders and those undergoing rehabilitation following musculoskeletal system injuries. Therefore, this specific property of swimming allows for the continuation of physical activity without further decline in pain and the development of further injury. It is specific for many target groups, such as elderly people and patients suffering from diseases such as osteoarthritis and rheumatoid arthritis. In fact, such groups have benefited from swimming in terms of joint function, pain reduction, and quality of life through its anti-inflammatory action.

It is also pointed out that swimming is beneficial in metabolic conditions, especially in complications arising from diabetes and obesity. In many studies, regular swimming exercises notably enhanced glucose metabolism, improved insulin sensitivity, and reduced the inflammatory burden. The beneficial effects observed in pancreatic tissues, including reduced levels of pro-inflammatory cytokines and enhanced insulin secretion, suggest that swimming may be a promising non-pharmacological intervention for managing diabetes and its complications. Furthermore, the effect of swimming on cardiovascular risk factors appeared promising, and it was further effective in improving lipid profile values, indicating a comprehensive exercise modality for enhancing metabolic health.

Despite the promising results, several limitations were observed in the existing literature. A remarkable proportion of studies in this review were conducted on animal models; therefore, results cannot be generalized to humans. There is also a need for long-term studies to assess the sustainability of the benefits observed for swimming in terms of its impact on inflammatory markers. Whereas most studies have focused on interventions that involved short periods, the length of time during which most positive results can be ascertained is challenging to achieve. Although the benefits of swimming have been observed for various states of health, the exact mechanisms by which it modulates inflammation remain to be deciphered. Future studies should be conducted to understand further the mechanisms in detail and the molecular pathways involved.

In addition, it highlights the importance of conducting more human studies that explicitly compare swimming’s relative effectiveness to other forms of exercise, such as cycling or running. For specific exercise recommendations tailored to individual needs, it is essential to understand the relative benefits of various forms of exercise. Additionally, the intensity and duration of the swimming intervention need to be identified for different health conditions in order to achieve maximum therapeutic effect with minimal adverse effects. Interestingly, in some cases, a high-intensity swimming regimen was less effective or even less effective than the moderate-intensity regimen. Thus, great caution is needed when prescribing swimming as a therapeutic exercise.

Thus, this comprehensive study can support the recommendation of swimming as a modality of exercise to modulate the inflammatory and anti-inflammatory responses in medical diseases. It is an ideal choice, as it represents a low-impact exercise that optimizes metabolic and immunological processes, particularly for individuals with chronic inflammatory disorders or those with limited mobility. However, if swimming therapy is to achieve its full therapeutic potential, more rigorous and well-designed human studies will be required. Long-term outcomes, optimal parameters of exercise, and comparative effectiveness will also need to be the focus of future studies to provide evidence-based guidelines that outline the incorporation of swimming into clinical and rehabilitative practice. Identifying and addressing these research gaps would better position swimming within comprehensive management strategies for inflammatory and chronic health conditions, thereby enhancing the outcomes and well-being of patients with these conditions.

## Figures and Tables

**Table 1:** Articles found by topic and relevant site.

	Searched Words + Swimming + Inflammatory	Databases	Total	Duplicate	Unrelated/Unavailable	Related/Citable
1	Alzheimer’s Disease	PubMed	36	22	98	7
		Scopus	91			
2	Parkinson	PubMed	9	7	39	6
		Scopus	43			
3	Neuropathies	PubMed	2	2	9	2
		Scopus	11			
4	Rheumatoid Arthritis	PubMed	6	5	11	3
		Scopus	13			
5	Gout	PubMed	1	1	2	1
		Scopus	3			
6	Osteoarthritis	PubMed	6	6	14	6
		Scopus	20			
7	Renal/kidney Disorders	PubMed	12	0	16	5
		Scopus	9			
8	Liver Disorders	PubMed	13	0	30	10
		Scopus	27			
9	Reproductive Disorders	PubMed	18	0	19	1
		Scopus	2			
10	Pancreatic Disorders	PubMed	2	1	5	5
		Scopus	9			
11	Respiratory Disorders	PubMed	8	1	21	4
		Scopus	18			
12	Diabetes	PubMed	24	10	74	26
		Scopus	86			
13	Cardiovascular Disorders	PubMed	24	3	10	22
		Scopus	11			
14	Negative effects of swimming		5	-	1	4

**Table 3:** Summary of results from human studies.

Row	Type of Disease	Subjects or Participants	Intervention	Results	**Reference**
1	Rheumatoid Arthritis	Women with Rheumatoid Arthritis	Swimming (3 times per week, 5 min warm-up, and 15 to 30 min specific exercises for lower limbs, which gradually increased) for 16 weeks.	Improvement in disease activity and functional ability in the water-based environment after 8 and 16 weeks. Improvement in disease activity, pain, and function	[94]
2	Respiratory Disorders	Competitive swimmers	Winter training Summer training	Winter training: ○ Connections between ↑ training hours per session = ↑ nasal congestion, rhinorrhea, and cough. In the summer group: ○ The same factors were not connected with respiratory symptoms.	[95]
3	Respiratory Disorders		Intense swimming training (ST)	↑ Airway mucosa eosinophil and mast cell count Greater submucosal type I and III collagen expression Higher mucin expression	[96]
4	Respiratory Disorders	Healthy nonsmoking adults aged 18–50, with no history of asthma or recent respiratory infection (past 3 weeks)	Swimming for 40 min	↑ Median serum Clara cell secretory protein levels Fractional exhaled nitric oxide was not altered overall but tended to be reduced among atopic individuals. There were no significant changes in lung function, surfactant protein D, 8-isoprostane, eight cytokines, or vascular endothelial growth factor.	[97]
5	Respiratory Disorders		Three years of swimming training	Skibinski’s cardio-respiratory index value ↑ is four times	[98]
6	Diabetes and Related Disorders	Diabetes mellitus type 2 patients	12 weeks of walking or running in a swimming pool (swimming group) or on a track (dry land group).	↓ Histone deacetylase activity ↑ Interlukin-10 levels	[99]
7	Diabetes and Related Disorders	Boys with type 1 diabetes	High-intensity Interval training (running and swimming) and resistance training 12 weeks, 3 days weekly	↓ Hemoglobin A1c and fasting blood sugar levels Improved VO_2_ peak, high-intensity lipoprotein cholesterol, and norepinephrine Cardiovascular health is enhanced through the improvement of autonomic modulation, VO_2 peak_, plasma lipids, and catecholamine levels	[100]
8	Cardiovascular Disorders	Middle-aged and older patients with osteoarthritis	swimming and cycling exercise Initially, 20 to 30 min/day and 3 days/week, 40% to 50% of the heart rate reserve intensity, which is elevated gradually to 40 to 45 min/day and 3 days/week at an intensity of 60% to 70% of heart rate	Both swimming and cycling training: ○ ↓ Central arterial stiffness and carotid artery stiffness ○ ↓ Interlukin-6 levels ○ No changes in other inflammatory markers after Swimming: ○ Elevated vascular endothelial function	[92]

↑: increase; ↓: decrease.

## Data Availability

No data were used to support the findings of this study.

## Abbreviations

6-OHDA	6-Hydroxydopamine
HSP70	70 kilodalton heat shock proteins
AD	Alzheimer’s disease
AMPK	AMP-activated protein kinase
AS	Ankylosing spondylitis
ADB	Anxiety and depression-like behavior
ApoE	Apolipoprotein E
Bcl-2	B-cell lymphoma-2
Bax	Bcl-2-associated X protein
BPA	Brachial plexus avulsion
BP	Blood pressure
BDNF	Brain-derived neurotrophic factor
BrdU	Bromodeoxyuridine
CREB	cAMP response element-binding protein
cTnI	Cardiac troponin I
CV	Cardiovascular
Cas-3	Caspase-3
CSF	Cerebrospinal fluid
CHF	Chronic heart failure
CC16	Clara cell protein
CWS	Cold-water swimming
CIA	Collagen-induced arthritis
CuNP	Copper nanoparticles
CRP	C-reactive protein
CKMB	Creatine kinase MB
cAMP	Cyclic adenosine monophosphate
DCM	Diabetic cardiomyopathy
DPN	Diarylpropionitrile
DBPs	Disinfection by-products
DA	Dopamine
DR2	Dopamine type 2 receptor
EA	Electroacupuncture
eNOS	Endothelial nitric oxide synthase
eWAT	Epididymal white adipose tissue
EMT	Epithelial-mesenchymal transition
EXE	Exercise
EAE	Experimental autoimmune encephalomyelitis
FBG	Fasting blood glucose
FATP-4	Fatty acid transport protein 4
FNDC5	Fibronectin type III domain-containing protein 5
FeNO	Fractional exhaled nitric oxide
FED	Fructose-enriched diet
GFAP	Glial fibrillary acidic protein
GLUT4	Glucose transporter type 4
GR	Glutathione reductase
GSH	Glutathione
GSK-3β	Glycogen synthase kinase-3 beta
HRR	Heart rate reserve
HRV	Heart rate variability
HDL	High-density lipoprotein
HFD	High-fat diet
HIIT	High-intensity interval training
HS	High-sodium
HDAC	Histone deacetylase
HDAC3	Histone deacetylase 3
HOMA-IR	Homeostatic model assessment of insulin resistance index
IgA	Immunoglobulin A
iNOS	Inducible nitric oxide synthase
I-κB	Inhibitor of kappa B
IR	Insulin resistance
IGF-1	Insulin-like Growth Factor-1
IFN-γ	Interferon gamma
IL-1β	Interleukin-1 beta
IRAK1	Interleukin-1 receptor-associated kinase 1
IL-10	Interleukin-10
IL-2	Interleukin-2
IL-6	Interleukin-6
IL-4	interleukin-4
ICV	Intracerebroventricular
ISO	Isoproterenol
LDH	Lactate dehydrogenase
LDL	Low-density lipoprotein
MΦ	Macrophages
MDA	Malondialdehyde
MMP-9	Matrix metalloproteinase-9
MMP-13	Matrix metalloproteinase-13
VO_2max_	Maximal oxygen uptake
MET	Metformin
MPTP	Mitochondrial permeability transition pore
MCP-1	Monocyte chemotactic protein-1
MS	Multiple sclerosis
MI	Myocardial infarction
NAC	N-acetylcysteine
NCAM	Neural cell adhesion molecule
NeuN	Neuronal nuclei
NAD	Nicotinamide adenine dinucleotide
NO	Nitric oxide
NAFLD	Nonalcoholic fatty liver disease
NSAIDs	Nonsteroidal anti-inflammatory drugs
ND	Normal diet
Nrf2	Nuclear factor erythroid 2-related factor 2
NF-κB	Nuclear factor kappa light-chain-enhancer of activated B lymphocytes
NOD	Non-obese diabetic
NLRP1	NLR family pyrin domain containing 1
NLRP3	NLR family pyrin domain containing 3
OA	Osteoarthritis
OVX	Ovariectomized
OS	Oxidative stress
PD	Parkinson’s disease
PLIN2	Perilipin-2
PPAR-γ	Peroxisome proliferator-activated receptor gamma
PPAR-α	Peroxisome proliferator-activated receptor alpha
PTX3	Pentraxin 3
PI3K	Phosphoinositide 3-kinase
PBMT	Photobiomodulation therapy
pIgR	Polymeric immunoglobulin receptor
PSD95	Postsynaptic density 95
PFN1	Profilin-1
PGC-1α	Proliferator-activated receptor gamma coactivator-1 alpha
PPT	Propyl pyrazole triol
Akt	Protein kinase B
RBC	Red blood cell
ROS	Reactive oxygen species
RANKL	Receptor activator of nuclear factor kappa-Β ligand
RVH	Renovascular hypertension
RA	Rheumatoid arthritis
sIgA	Secretory immunoglobulin A
SCD1	Severe combined immunodeficiency 1
SG	Sebaceous gland
SEMA3A	Semaphorin 3A
SS	Serotonergic system
SIRT1	Sirtuin 1
SCRI	Skibinski’s cardio-respiratory index
STZ	Streptozotocin
SOD	Superoxide dismutase
ST	Swimming training
SNS	Sympathetic nervous system
sWAT	Substance white adipose tissue
SP-D	Surfactant protein D
SBP	Systolic blood pressure
TRAF6	TNF receptor-associated factor 6
TLR-4	Toll-like receptor 4
TCA	Total antioxidant capacity
TNF-α	Tumor necrosis factor alpha
T1D	Type 1 diabetes
T2D	Type 2 diabetes
T2DM	Type 2 diabetes mellitus
VEGF	Vascular endothelial growth factor
ZDF	Zucker diabetic fatty
α7nAChR	α7nicotinic acetylcholine receptor
Aβ	β-Amyloid peptide

## References

[1] Wei W., Lin Z., Xu P., Lv X., Lin L., Li Y., Zhou Y., Lu T., Xue X. (2023). Diet Control and Swimming Exercise Ameliorate HFD-Induced Cognitive Impairment Related to the SIRT1-NF-κB/PGC-1α Pathways in ApoE^−/−^ Mice. Neural Plast..

[2] Oja P., Memon A.R., Titze S., Jurakic D., Chen S.-T., Shrestha N., Em S., Matolic T., Vasankari T., Heinonen A. (2024). Health Benefits of Different Sports: A Systematic Review and Meta-Analysis of Longitudinal and Intervention Studies Including 2.6 Million Adult Participants. Sports Med.-Open.

[3] Trinidad A., González-Garcia H., López-Valenciano A. (2021). An Updated Review of the Epidemiology of Swimming Injuries. PMR.

[4] Mahdirejei H.A., Peeri M., Azarbayjani M.A., Fattahi Masrour F. (2023). Fluoxetine combined with swimming exercise synergistically reduces lipopolysaccharide-induced depressive-like behavior by normalizing the HPA axis and brain inflammation in mice. Pharmacol. Biochem. Behav..

[5] Shekarchian M., Peeri M., Azarbayjani M.A. (2023). Physical activity in a swimming pool attenuates memory impairment by reducing glutamate and inflammatory cytokines and increasing BDNF in the brain of mice with type 2 diabetes. Brain Res. Bull..

[6] Aghaei F., Wong A., Zargani M., Sarshin A., Feizolahi F., Derakhshan Z., Hashemi M., Arabzadeh E. (2023). Effects of swimming exercise combined with silymarin and vitamin C supplementation on hepatic inflammation, oxidative stress, and histopathology in elderly rats with high-fat diet-induced liver damage. Nutrition.

[7] Goto S., Radák Z., Nyakas C., Hae Y.C., Naito H., Takahashi R., Nakamoto H., Abe R. (2004). Regular exercise: An effective means to reduce oxidative stress in old rats. Ann. N. Y. Acad. Sci..

[8] Rahmanian-Devin P., Rakhshandeh H., Baradaran Rahimi V., Sanei-Far Z., Hasanpour M., Memarzia A., Iranshahi M., Askari V.R. (2021). Intraperitoneal Lavage with Crocus sativus Prevents Postoperative-Induced Peritoneal Adhesion in a Rat Model: Evidence from Animal and Cellular Studies. Oxid. Med. Cell. Longev..

[9] Yahyazadeh R., Baradaran Rahimi V., Yahyazadeh A., Mohajeri S.A., Askari V.R. (2021). Promising effects of gingerol against toxins: A review article. Biofactors.

[10] Bosak F., Baradaran Rahimi V., Sobhani B., Dabbaghi M.M., Soukhtanloo M., Zahedi Avval F., Askari V.R. (2025). Evaluation of the Protective Effects of Noscapine on Paraquat-Induced Parkinson’s Disease in Rats. Mol. Neurobiol..

[11] Baradaran Rahimi V., Askari V.R., Hosseini M., Yousefsani B.S., Sadeghnia H.R. (2019). Anticonvulsant Activity of Viola tricolor against Seizures Induced by Pentylenetetrazol and Maximal Electroshock in Mice. Iran. J. Med. Sci..

[12] Alamri H.S., Mufti R., Sabir D.K., Abuderman A.A., Dawood A.F., ShamsEldeen A.M., Haidara M.A., Isenovic E.R., El-Bidawy M.H. (2023). Forced Swimming-Induced Depressive-like Behavior and Anxiety Are Reduced by Chlorpheniramine via Suppression of Oxidative and Inflammatory Mediators and Activating the Nrf2-BDNF Signaling Pathway. Curr. Issues Mol. Biol..

[13] Askari V.R., Baradaran Rahimi V., Assaran A., Iranshahi M., Boskabady M.H. (2020). Evaluation of the anti-oxidant and anti-inflammatory effects of the methanolic extract of Ferula szowitsiana root on PHA-induced inflammation in human lymphocytes. Drug Chem. Toxicol..

[14] Akhlaghipour I., Shad A.N., Askari V.R., Maharati A., Rahimi V.B. (2023). How caffeic acid and its derivatives combat diabetes and its complications: A systematic review. J. Funct. Foods.

[15] Yoshida S., Hagiwara Y., Tsuchiya M., Shinoda M., Koide M., Hatakeyama H., Chaweewannakorn C., Suzuki K., Yano T., Sogi Y. (2019). Involvement of inflammasome activation via elevation of uric acid level in nociception in a mouse model of muscle pain. Mol. Pain.

[16] Askari V.R., Khosravi K., Baradaran Rahimi V., Garzoli S. (2024). A Mechanistic Review on How Berberine Use Combats Diabetes and Related Complications: Molecular, Cellular, and Metabolic Effects. Pharmaceuticals.

[17] Rakhshandeh H., Rajabi Khasevan H., Saviano A., Mahdinezhad M.R., Baradaran Rahimi V., Ehtiati S., Etemad L., Ebrahimzadeh-Bideskan A., Maione F., Askari V.R. (2022). Protective Effect of Portulaca oleracea on Streptozotocin-Induced Type I Diabetes-Associated Reproductive System Dysfunction and Inflammation. Molecules.

[18] Salem H.A., Abu-Elfotuh K., Alzahrani S., Rizk N.I., Ali H.S., Elsherbiny N., Aljohani A., Hamdan A.M.E., Chellasamy P., Abdou N.S. (2023). Punicalagin’s Protective Effects on Parkinson’s Progression in Socially Isolated and Socialized Rats: Insights into Multifaceted Pathway. Pharmaceutics.

[19] Che Ramli M.D.B., Nizam N.B.B., Uzid M.B.M., Md Nazrey N.A.B., Mazlan N.B., Mohd Mizan N.B., Muhammad H., Hasan M.K.N. (2022). The therapeutic effect of bacopa monnieri in treating parkinson’s disease. Int. J. Med. Toxicol. Leg. Med..

[20] Ding Z., Du L. (2022). Swimming exercise ameliorates depressive-like behavior by anti-inflammation activity, rebalancing gut Escherichia coli and Lactobacilli. Brain Res..

[21] Farsani M.S., Fathi M., Farsani Z.H., Gourgin Karaji Z. (2024). Swimming alters some proteins of skeletal muscle tissue in rats with Alzheimer-like phenotype. Arch. Gerontol. Geriatr..

[22] Xie Y., Wu Z., Sun L., Zhou L., Xiao L., Wang H., Wang G. (2022). Swimming exercise reverses chronic unpredictable mild stress-induced depression-like behaviors and alleviates neuroinflammation and collapsing response mediator protein-2-mediated neuroplasticity injury in adult male mice. NeuroReport.

[23] Liu Y., Meng X.K., Shao W.Z., Liu Y.Q., Tang C., Deng S.S., Tang C.F., Zheng L., Guo W. (2024). miR-34a/TAN1/CREB Axis Engages in Alleviating Oligodendrocyte Trophic Factor-Induced Myelin Repair Function and Astrocyte-Dependent Neuroinflammation in the Early Stages of Alzheimer’s Disease: The Anti-Neurodegenerative Effect of Treadmill Exercise. Neurochem. Res..

[24] Karaji Z.G., Fathi M., Mirnasori R., van der Zee E.A. (2023). Swimming exercise and clove oil can improve memory by molecular responses modification and reduce dark cells in rat model of Alzheimer’s disease. Exp. Gerontol..

[25] Liu Z.T., Ma Y.T., Pan S.T., Xie K., Shen W., Lin S.Y., Gao J.Y., Li W.Y., Li G.Y., Wang Q.W. (2022). Effects of involuntary treadmill running in combination with swimming on adult neurogenesis in an Alzheimer’s mouse model. Neurochem. Int..

[26] Hegazy M.A., Abdelmonsif D.A., Zeitoun T.M., El-Sayed N.S., Samy D.M. (2022). Swimming exercise versus L-carnosine supplementation for Alzheimer’s dementia in rats: Implication of circulating and hippocampal FNDC5/irisin. J. Physiol. Biochem..

[27] Bashiri H., Enayati M., Bashiri A., Salari A.A. (2020). Swimming exercise improves cognitive and behavioral disorders in male NMRI mice with sporadic Alzheimer-like disease. Physiol. Behav..

[28] Wu C., Yang L., Tucker D., Dong Y., Zhu L., Duan R., Liu T.C., Zhang Q. (2018). Beneficial Effects of Exercise Pretreatment in a Sporadic Alzheimer’s Rat Model. Med. Sci. Sports Exerc..

[29] Gergin S., Kirazlı Ö., Boracı H., Yıldız S.D., Yananlı H.R., Şehirli Ü.S. (2023). The effects of regular swimming exercise and melatonin on the neurons localized in the striatum of hemiparkinsonian rats. Anat. Sci. Int..

[30] Skripkina N.A., Smolentseva I.G., Kuzmina A.V., Levin O.S. (2021). Unusual failure of swimming skills in patients with Parkinson’s disease. Zhurnal Nevrologii i Psikhiatrii Imeni SS Korsakova.

[31] Neves M.A., Bouça-Machado R., Guerreiro D., Caniça V., Pona-Ferreira F., Ferreira J.J. (2020). Swimming is compromised in Parkinson’s disease patients. Mov. Disord..

[32] Boracı H., Kirazlı Ö., Gülhan R., Yıldız Sercan D., Şehirli Ü.S. (2020). Neuroprotective effect of regular swimming exercise on calretinin-positive striatal neurons of Parkinsonian rats. Anat. Sci. Int..

[33] Ali A.A., Abo El-Ella D.M., El-Emam S.Z., Shahat A.S., El-Sayed R.M. (2019). Physical & mental activities enhance the neuroprotective effect of vinpocetine & coenzyme Q10 combination against Alzheimer & bone remodeling in rats. Life Sci..

[34] Hsieh Y.L., Yang N.P., Chen S.F., Lu Y.L., Yang C.C. (2022). Early Intervention of Cold-Water Swimming on Functional Recovery and Spinal Pain Modulation Following Brachial Plexus Avulsion in Rats. Int. J. Mol. Sci..

[35] Debastiani J.C., Santana A.J., Ribeiro L.D.F.C., Brancalhão R.M.C., Bertolini G.R.F. (2019). Sericin silk protein in peripheral nervous repair associated with the physical exercise of swimming in Wistar rats. Neurol. Res..

[36] Goes A.T.R., Souza L.C., Filho C.B., Del Fabbro L., De Gomes M.G., Boeira S.P., Jesse C.R. (2014). Neuroprotective effects of swimming training in a mouse model of Parkinson’s disease induced by 6-hydroxydopamine. Neuroscience.

[37] Zhu S.J., Yang Y.T., Cao Y.T., Zheng L.D., Lin K.L., Zhu R. (2024). Cartilage protective effect of swimming exercise in aged mice with knee osteoarthritis. Chin. J. Tissue Eng. Res..

[38] Saber M.M., Mahmoud M.M., Amin H.M., Essam R.M. (2023). Therapeutic effects of combining curcumin and swimming in osteoarthritis using a rat model. Biomed. Pharmacother..

[39] Martins G.A., Degen A.N., Antunes F.T.T., da Rosa L.G., Ferraz A.G., Wiilland E., Vieira L.B., de Souza A.H. (2022). Benefits of electroacupuncture and a swimming association when compared with isolated protocols in an osteoarthritis model. J. Tradit. Complement. Med..

[40] da Silva L.A., Thirupathi A., Colares M.C., Haupenthal D.P.D.S., Venturini L.M., Corrêa M.E.A.B., Silveira G.D.B., Haupenthal A., do Bomfim F.R.C., de Andrade T.A.M. (2023). The effectiveness of treadmill and swimming exercise in an animal model of osteoarthritis. Front. Physiol..

[41] Tomazoni S.S., Leal-Junior E.C., Frigo L., Pallotta R.C., Teixeira S., de Almeida P., Bjordal J.M., Lopes-Martins R. (2016). Isolated and combined effects of photobiomodulation therapy, topical nonsteroidal anti-inflammatory drugs, and physical activity in the treatment of osteoarthritis induced by papain. J. Biomed. Opt..

[42] Navarro F., Bacurau A.V., Almeida S.S., Barros C.C., Moraes M.R., Pesquero J.L., Ribeiro S.M., Araújo R.C., Costa Rosa L.F., Bacurau R.F. (2010). Exercise prevents the effects of experimental arthritis on the metabolism and function of immune cells. Cell Biochem. Funct..

[43] Shi L., Xu L., Yin L., Zeng F.W., Mei Q. (2012). Effect of swimming in cold water on gouty arthritis of rats with hyperuricemia. Chin. J. Pharmacol. Toxicol..

[44] Zhao H.X., Zhang Z., Hu F., Wei Q.F., Yu Y.S., Zhao H.D. (2023). Swimming exercise activates peroxisome proliferator-activated receptor-alpha and mitigates age-related renal fibrosis in rats. Mol. Cell. Biochem..

[45] de Souza J.A., Becker L.K., Batista M.A.C., de Assis Braga D.C., Gomes P.M., Alzamora A.C., Vieira M.A.R., de Lima W.G., Andrade M.G.C., de Lima Sanches B. (2021). Swimming training improves cardiovascular autonomic dysfunctions and prevents renal damage in rats fed a high-sodium diet from weaning. Exp. Physiol..

[46] Farzanegi P., Asadi M., Abdi A., Etemadian M., Amani M., Amrollah V., Shahri F., Gholami V., Abdi Z., Moradi L. (2020). Swimming exercise in combination with garlic extract administration as a therapy against doxorubicin-induced hepatic, heart and renal toxicity to rats. Toxin Rev..

[47] Kumral Z.N., Sener G., Ozgur S., Koc M., Suleymanoglu S., Hurdag C., Yegen B.C. (2016). Regular exercise alleviates renovascular hypertension-induced cardiac/endothelial dysfunction and oxidative injury in rats. J. Physiol. Pharmacol..

[48] Bernardes D., Oliveira-Lima O.C., da Silva T.V., Juliano M.A., dos Santos D.M., Carvalho-Tavares J. (2016). Metabolic Alterations in Experimental Autoimmune Encephalomyelitis in Mice: Effects of Prior Physical Exercise. Neurophysiology.

[49] Nazari M., Kordi M.R., Minasian V., Quchan A.H.S.K. (2022). Ameliorating effect of 6-week swimming exercise on mice with experimental autoimmune encephalomyelitis (EAE) by reducing fetuin-A and increasing AMPK & NAD+ levels in liver tissue. Iran. J. Basic Med. Sci..

[50] Huang W.C., Xu J.W., Li S., Ng X.E., Tung Y.T. (2022). Effects of exercise on high-fat diet-induced non-alcoholic fatty liver disease and lipid metabolism in ApoE knockout mice. Nutr. Metab..

[51] Godínez-Victoria M., Drago-Serrano M.E., Reyna-Garfias H., Viloria M., Lara-Padilla E., Resendiz-Albor A.A., Sánchez-Torres L.E., Cruz-Hernández T.R., Campos-Rodriguez R. (2012). Effects on secretory IgA levels in small intestine of mice that underwent moderate exercise training followed by a bout of strenuous swimming exercise. Brain Behav. Immun..

[52] Schultz A., Mendonca L.S., Aguila M.B., Mandarim-de-Lacerda C.A. (2012). Swimming training beneficial effects in a mice model of nonalcoholic fatty liver disease. Exp. Toxicol. Pathol..

[53] Zhang Q.B., Zhang B.H., Zhang K.Z., Meng X.T., Jia Q.A., Zhang Q.B., Bu Y., Zhu X.D., Ma D.N., Ye B.G. (2016). Moderate swimming suppressed the growth and metastasis of the transplanted liver cancer in mice model: With reference to nervous system. Oncogene.

[54] Goto S., Radák Z. (2007). Regular exercise attenuates oxidative stress in aging rat tissues: A possible mechanism toward anti-aging medicine. J. Exerc. Sci. Fit..

[55] Zhang Y., Xu J., Zhou D., Ye T., Zhou P., Liu Z., Liu X., Wang Z., Hua T., Zhang Z. (2023). Swimming exercise ameliorates insulin resistance and nonalcoholic fatty liver by negatively regulating PPARγ transcriptional network in mice fed high fat diet. Mol. Med..

[56] Altintas F., Caliskan S., Ozmen O., Kilic-Toprak E. (2022). Swimming exercise restores damaging effects of fructose-enriched diet on the liver in rats. Tissue Cell.

[57] Kolieb E., Maher S.A., Shalaby M.N., Alsuhaibani A.M., Alharthi A., Hassan W.A., El-Sayed K. (2022). Vitamin D and Swimming Exercise Prevent Obesity in Rats under a High-Fat Diet via Targeting FATP4 and TLR4 in the Liver and Adipose Tissue. Int. J. Environ. Res. Public Health.

[58] Yi X., Tang D., Cao S., Li T., Gao H., Ma T., Yao T., Li J., Chang B. (2020). Effect of different exercise loads on testicular oxidative stress and reproductive function in obese male mice. Oxid. Med. Cell. Longev..

[59] Habibi P., Ahmadiasl N., Nourazarian A., Yousefi H. (2022). Swimming exercise improves SIRT1, NF-κB, and IL-1β protein levels and pancreatic tissue injury in ovariectomized diabetic rats. Horm. Mol. Biol. Clin. Investig..

[60] Alipour M.R., Yousefzade N., Bavil F.M., Naderi R., Ghiasi R. (2020). Swimming impacts on pancreatic inflammatory cytokines, mir-146a and NF-κB expression levels in type-2 diabetic rats. Curr. Diabetes Rev..

[61] Elbassuoni E.A., Abdel Hafez S.M. (2019). Impact of chronic exercise on counteracting chronic stress-induced functional and morphological pancreatic changes in male albino rats. Cell Stress Chaperones.

[62] Ghiasi R., Soufi F.G., Mohaddes G., Alihemmati A., Somi M.H., Ebrahimi H., Bavil F.M., Alipour M.R. (2016). Influance of regular swimming on serum levels of CRP, IL-6, TNF-α in high-fat diet-induced type 2 diabetic rats. Gen. Physiol. Biophys..

[63] Teixeira de Lemos E., Reis F., Baptista S., Pinto R., Sepodes B., Vala H., Rocha-Pereira P., Correia da Silva G., Teixeira N., Silva A.S. (2009). Exercise training decreases proinflammatory profile in Zucker diabetic (type 2) fatty rats. Nutrition.

[64] Özüdoğru E., Atay E., Savran M., Aşci H., Özmen Ö., Topsakal Ş. (2023). Protective effects of swimming exercises and metformin on cardiac and aortic damage caused by a high-fat diet in obese rats with type 2 diabetes, by regulating the Bcl2/Bax signaling pathway. Turk. J. Med. Sci..

[65] Guo Y., Zhang Q., Zheng L., Shou J., Zhuang S., Xiao W., Chen P. (2023). Depot-specific adaption of adipose tissue for different exercise approaches in high-fat diet/streptozocin-induced diabetic mice. Front. Physiol..

[66] Azizi N., Rahbarghazi A., Bavil F.M., Rahbarghazi R., Ghaffari-Nasab A., Rezaie J., Delkhosh A., Ahmadi M. (2023). Swimming training reduced inflammation and apoptotic changes in pulmonary tissue in type 1 diabetic mice. J. Diabetes Metab. Disord..

[67] Ya L., Xia L., Penghui D., Wei J., Jianping L. (2022). Exercise effects on myocardial type I, III collagen and angiotensin II/transforming growth factor beta1/Smad2 pathway in diabetic myocardial fibrosis rats. Chin. J. Tissue Eng. Res..

[68] Eldesoqui M., Eldken Z.H., Mostafa S.A., Al-Serwi R.H., El-Sherbiny M., Elsherbiny N., Mohammedsaleh Z.M., Sakr N.H. (2022). Exercise Augments the Effect of SGLT2 Inhibitor Dapagliflozin on Experimentally Induced Diabetic Cardiomyopathy, Possible Underlying Mechanisms. Metabolites.

[69] Daghigh F., Karimi P., Alihemmati A., Majidi Zolbin M., Ahmadiasl N. (2022). Swimming training modulates lung injury induced by ovariectomy in diabetic rats: Involvement of inflammatory and fibrotic biomarkers. Arch. Physiol. Biochem..

[70] da Silva Pereira M.M., de Melo I.M.F., Braga V.A.Á., Teixeira Á.A.C., Wanderley-Teixeira V. (2022). Effect of swimming exercise, insulin-associated or not, on inflammatory cytokines, apoptosis, and collagen in diabetic rat placentas. Histochem. Cell Biol..

[71] Sadeghian R., Shahidi S., Komaki A., Habibi P., Ahmadiasl N., Yousefi H., Daghigh F. (2021). Synergism effect of swimming exercise and genistein on the inflammation, oxidative stress, and VEGF expression in the retina of diabetic-ovariectomized rats. Life Sci..

[72] Rahman M.M., Park S.J., Jeon H.Y., Kim S. (2021). Exercise and oral melatonin attenuate anxiety and depression like behavior in type 2 diabetic rats. J. Adv. Biotechnol. Exp. Ther..

[73] Netto R.O.R.F., Moura E.G., Ferretti R., de Andrade Araújo M.J., Mâncio R.D., da Silva R.E., Cajazeiro D.C., Fernandes V.A.R., Bortolato R.S., Col L.O. (2021). Combine treatment with N-acetylcysteine, anti-CD4/CD8 antibodies and physical exercise reduces histopathological damage in salivary glands of spontaneously diabetic mice. Rom. J. Diabetes Nutr. Metab. Dis..

[74] Gilak-Dalasm M., Peeri M., Azarbayjani M.A. (2021). Swimming exercise decreases depression-like behaviour and inflammatory cytokines in a mouse model of type 2 diabetes. Exp. Physiol..

[75] Chen Z., Qin X., Zhang X., Liu B., Chen M. (2020). Upregulation of IL-4 signaling contributes to aerobic exercise-induced insulin sensitivity. Biochem. Biophys. Res. Commun..

[76] Montenegro M.L., Bonocher C.M., Meola J., Portella R.L., Ribeiro-Silva A., Brunaldi M.O., Ferriani R.A., Rosa-e-Silva J.C. (2019). Effect of Physical Exercise on Endometriosis Experimentally Induced in Rats. Reprod. Sci..

[77] Rahman M.M., Kwon H.S., Kim M.J., Go H.K., Oak M.H., Kim D.H. (2017). Melatonin supplementation plus exercise behavior ameliorate insulin resistance, hypertension and fatigue in a rat model of type 2 diabetes mellitus. Biomed. Pharmacother..

[78] Motta V.F., Aguila M.B., Mandarim-De-lacerda C.A. (2016). High-intensity interval training (swimming) significantly improves the adverse metabolism and comorbidities in diet-induced obese mice. J. Sports Med. Phys. Fit..

[79] Kesherwani V., Chavali V., Hackfort B.T., Tyagi S.C., Mishra P.K. (2015). Exercise ameliorates high fat diet induced cardiac dysfunction by increasing interleukin 10. Front. Physiol..

[80] Teixeira De Lemos E., Pinto R., Oliveira J., Garrido P., Sereno J., Mascarenhas-Melo F., Páscoa-Pinheiro J., Teixeira F., Reis F. (2011). Differential effects of acute (extenuating) and chronic (training) exercise on inflammation and oxidative stress status in an animal model of type 2 diabetes mellitus. Mediat. Inflamm..

[81] Teixeira De Lemos E.T., Reis F., Baptista S., Pinto R., Sepodes B., Vala H., Rocha-Pereira P., Silva A.S., Teixeira F. (2007). Exercise training is associated with improved levels of C-reactive protein and adiponectin in ZDF (type 2) diabetic rats. Med. Sci. Monit..

[82] Sharma A.K., Kumar A., Taneja G., Nagaich U., Deep A., Datusalia A.K., Rajput S.K. (2018). Combined and individual strategy of exercise generated preconditioning and low dose copper nanoparticles serve as superlative approach to ameliorate ISO-induced myocardial infarction in rats. Pharmacol. Rep..

[83] Chen W.K., Tsai Y.L., Shibu M.A., Shen C.Y., Chang-Lee S.N., Chen R.J., Yao C.H., Ban B., Kuo W.W., Huang C.Y. (2018). Exercise training augments Sirt1-signaling and attenuates cardiac inflammation in D-galactose induced-aging rats. Aging.

[84] Bulut E.C., Abueid L., Ercan F., Süleymanoğlu S., Ağırbaşlı M., Yeğen B. (2016). Treatment with oestrogen-receptor agonists or oxytocin in conjunction with exercise protects against myocardial infarction in ovariectomized rats. Exp. Physiol..

[85] Silva D.B., Miranda A.P., Silva D.B., D’Angelo L.R., Rosa B.B., Soares E.A., Ramalho J.G., Boriollo M.F., Garcia J.A. (2015). Propolis and swimming in the prevention of atherogenesis and left ventricular hypertrophy in hypercholesterolemic mice. Braz. J. Biol..

[86] Preto E., Lima N.E., Simardi L., Fonseca F.L., Filho A.A., Maifrino L.B. (2015). Effect of mild aerobic training on the myocardium of mice with chronic Chagas disease. Biol. Targets Ther..

[87] Nounou H.A., Deif M.M., Shalaby M.A. (2012). Effect of flaxseed supplementation and exercise training on lipid profile, oxidative stress and inflammation in rats with myocardial ischemia. Lipids Health Dis..

[88] Baptista S., Piloto N., Reis F., Teixeira-De-Lemos E., Garrido A.P., Dias A., Lourenço M., Palmeiro A., Ferrer-Antunes C., Teixeira F. (2008). Treadmill running and swimming imposes distinct cardiovascular physiological adaptations in the rat: Focus on serotonergic and sympathetic nervous systems modulation. Acta Physiol. Hung..

[89] Nunes R.B., Tonetto M., Machado N., Chazan M., Heck T.G., Veiga A.B., Dall’Ago P. (2008). Physical exercise improves plasmatic levels of IL-10, left ventricular end-diastolic pressure, and muscle lipid peroxidation in chronic heart failure rats. J. Appl. Physiol..

[90] Kuzmina A.V., Smolentseva I.G., Levin O.S. (2022). Swimming disorders in Parkinson’s disease. Zhurnal Nevrologii i Psikhiatrii Imeni SS Korsakova.

[91] Yázigi F., Espanha M., Vieira F., Messier S.P., Monteiro C., Veloso A.P. (2013). The PICO project: Aquatic exercise for knee osteoarthritis in overweight and obese individuals. BMC Musculoskelet. Disord..

[92] Alkatan M., Machin D.R., Baker J.R., Akkari A.S., Park W., Tanaka H. (2016). Effects of Swimming and Cycling Exercise Intervention on Vascular Function in Patients With Osteoarthritis. Am. J. Cardiol..

[93] Ibrahim Y.M.A.E., Othman G. (2014). Challenge of diabetes control in patients with rheumatic diseases. Kuwait Med. J..

[94] Siqueira U.S., Orsini Valente L.G., de Mello M.T., Szejnfeld V.L., Pinheiro M.M. (2017). Effectiveness of Aquatic Exercises in Women With Rheumatoid Arthritis: A Randomized, Controlled, 16-Week Intervention-The HydRA Trial. Am. J. Phys. Med. Rehabil..

[95] Zaccarin M., Zanni S., Gallè F., Protano C., Valeriani F., Liguori G., Romano Spica V., Vitali M. (2022). Studying Respiratory Symptoms Related to Swimming Pools Attendance in Young Athletes: The SPHeRA Study. Toxics.

[96] Bougault V., Loubaki L., Joubert P., Turmel J., Couture C., Laviolette M., Chakir J., Boulet L.P. (2012). Airway remodeling and inflammation in competitive swimmers training in indoor chlorinated swimming pools. J. Allergy Clin. Immunol..

[97] Font-Ribera L., Kogevinas M., Zock J., Gómez F.P., Barreiro E., Nieuwenhuijsen M.J., Fernandez P., Lourencetti C., Pérez-Olabarría M., Bustamante M. (2010). Short-term changes in respiratory biomarkers after swimming in a chlorinated pool. Environ. Health Perspect..

[98] Pachalski A., Mekarski T. (1980). Effect of swimming on increasing of cardio-respiratory capacity in paraplegics. Paraplegia.

[99] Korb A., Bertoldi K., Lovatel G.A., Delevatti R.S., Elsner V.R., Meireles L.C.F., Kruel L.F.M., Siqueira I.R. (2018). Acute exercise and periodized training in different environments affect histone deacetylase activity and interleukin-10 levels in peripheral blood of patients with type 2 diabetes. Diabetes Res. Clin. Pract..

[100] Saki H., Nazem F., Fariba F., Sheikhsharbafan R. (2023). A High intensity Interval training (running and swimming) and resistance training intervention on heart rate variability and the selected biochemical factors in boys with type 1 diabetes. Diabetes Res. Clin. Pract..

[101] Rohm T.V., Meier D.T., Olefsky J.M., Donath M.Y. (2022). Inflammation in obesity, diabetes, and related disorders. Immunity.

[102] Santos G.R., Cunha M.R., Caldeira E.J., Galdeano E.A., Prudente R.C.S., Pinto C.A.L. (2021). Effect of antioxidant treatment with n-acetylcysteine and swimming on lipid expression of sebaceous glands in diabetic mice. Sci. Rep..

[103] Tso J., Hollowed C., Liu C., Alkhoder A., Dommisse M., Gowani Z., Miller A., Nguyen G., Nguyen P., Prabakaran G. (2020). Nonsteroidal Anti-inflammatory Drugs and Cardiovascular Risk in American Football. Med. Sci. Sports Exerc..

[104] Santos M.H., Higuchi Mde L., Tucci P.J., Garavelo S.M., Reis M.M., Antonio E.L., Serra A.J., Maranhão R.C. (2016). Previous exercise training increases levels of PPAR-α in long-term post-myocardial infarction in rats, which is correlated with better inflammatory response. Clinics.

[105] Shapoval L.N., Pobegailo L.S., Stepanenko L.G., Dmytrenko O.V., Bouryi V.A., Sagach V.F. (2011). Impact of swimming exercise training on the effects of modulation of mitochondrial permeability transition and NOS-1 activation in medullary cardiovascular neurons of rats. Neurophysiology.

[106] Manolis A.S., Manolis S.A., Manolis A.A., Manolis T.A., Apostolaki N., Melita H. (2019). Winter swimming: Body hardening and cardiorespiratory protection via sustainable acclimation. Curr. Sports Med. Rep..

[107] Llorens-Martín M., Jurado-Arjona J., Bolós M., Pallas-Bazarra N., Ávila J. (2016). Forced swimming sabotages the morphological and synaptic maturation of newborn granule neurons and triggers a unique pro-inflammatory milieu in the hippocampus. Brain Behav. Immun..

[108] Gatmaitan B.G., Chason J.L., Lerner A.M. (1970). Augmentation of the virulence of murine coxsackie-virus B-3 myocardiopathy by exercise. J. Exp. Med..

[109] Lombardi G., Sanchis-Gomar F., Perego S., Sansoni V., Banfi G. (2016). Implications of exercise-induced adipo-myokines in bone metabolism. Endocrine.

[110] Koop M.A., Sleijser-Koehorst M.L.S., Hooijmans C.R., Tdlohreg P.Q., Lutke Schipholt I.J., Scholten-Peeters G.G.M., Coppieters M.W. (2023). The potential protective effects of pre-injury exercise on neuroimmune responses following experimentally-induced traumatic neuropathy: A systematic review with meta-analysis. Front. Immunol..

[111] Da Silva Santos R., Galdino G. (2018). Endogenous systems involved in exercise-induced analgesia. J. Physiol. Pharmacol..

[112] Oh S.J., Ahn H., Jung K.H., Han S.J., Nam K.R., Kang K.J., Park J.A., Lee K.C., Lee Y.J., Choi J.Y. (2020). Evaluation of the Neuroprotective Effect of Microglial Depletion by CSF-1R Inhibition in a Parkinson’s Animal Model. Mol. Imaging Biol..

[113] Becker B.E. (2009). Aquatic therapy: Scientific foundations and clinical rehabilitation applications. PM&R.

[114] Faíl L.B., Marinho D.A., Marques E.A., Costa M.J., Santos C.C., Marques M.C., Izquierdo M., Neiva H.P. (2022). Benefits of aquatic exercise in adults with and without chronic disease-A systematic review with meta-analysis. Scand. J. Med. Sci. Sports.

[115] Mooventhan A., Nivethitha L. (2014). Scientific evidence-based effects of hydrotherapy on various systems of the body. N. Am. J. Med. Sci..

[116] Cole A.J., Becker B.E. (2004). Comprehensive Aquatic Therapy.

[117] Font-Ribera L., Villanueva C.M., Nieuwenhuijsen M.J., Zock J.P., Kogevinas M., Henderson J. (2011). Swimming pool attendance, asthma, allergies, and lung function in the Avon Longitudinal Study of Parents and Children cohort. Am. J. Respir. Crit. Care Med..

[118] Bougault V., Turmel J., St-Laurent J., Bertrand M., Boulet L.P. (2009). Asthma, airway inflammation and epithelial damage in swimmers and cold-air athletes. Eur. Respir. J..

